# Pyrazolo[4,3-*e*]tetrazolo[1,5-*b*][1,2,4]triazine Sulfonamides as Novel Potential Anticancer Agents: Cytotoxic and Genotoxic Activities In Vitro

**DOI:** 10.3390/molecules27123761

**Published:** 2022-06-11

**Authors:** Karol Bukowski, Beata Marciniak, Mateusz Kciuk, Mariusz Mojzych, Renata Kontek

**Affiliations:** 1 Department of Molecular Biotechnology and Genetics, Faculty of Biology and Environmental Protection, University of Lodz, 12/16 Banacha St., 90-237 Lodz, Poland; beata.marciniak@biol.uni.lodz.pl (B.M.); mateusz.kciuk@edu.uni.lodz.pl (M.K.); renata.kontek@biol.uni.lodz.pl (R.K.); 2 Doctoral School of Exact and Natural Sciences, University of Lodz, 12/16 Banacha St., 90-237 Lodz, Poland; 3 Department of Chemistry, Siedlce University of Natural Sciences and Humanities, 3 Maja 54, 08-110 Siedlce, Poland; mariusz.mojzych@uph.edu.pl

**Keywords:** pyrazolo[4,3-*e*]tetrazolo[1,5-*b*][1,2,4]triazine, sulfonamides, anticancer agents, cancer cells, PBMCs, cytotoxicity, genotoxicity, MTT, comet assay, γ-H2AX

## Abstract

In this paper, we present for the first time the evaluation of cytotoxicity and genotoxicity of de novo synthesized pyrazolo[4,3-*e*]tetrazolo[1,5-*b*][1,2,4]triazine sulfonamides **MM129**, **MM130**, and **MM131** in human tumor cell lines: HeLa, HCT 116, PC-3, and BxPC-3. Cytotoxic and genotoxic properties of the tested compounds were estimated using the MTT assay, comet assay (alkaline and neutral version), and γ-H2AX immuno-staining. Examined sulfonamides exhibited strong anticancer properties towards tested cells in a very low concentration range (IC_50_ = 0.17–1.15 μM) after 72 h exposure time. The results of the alkaline and neutral version of the comet assay following 24 h incubation of the cells with tested compounds demonstrated the capability of heterocycles to induce significant DNA damage in exposed cells. HCT 116 cells were the most sensitive to the genotoxic activity of novel tricyclic pyrazolo[4,3-*e*]tetrazolo[1,5-*b*][1,2,4]triazine sulfonamides in the neutral version of the comet assay. Immunocytochemical detection of γ-H2AX showed an increase in DNA DSBs level in the HCT 116 cell line, after 24 h incubation with all tested compounds, confirming the results obtained in the neutral comet assay. Among all investigated compounds, **MM131** showed the strongest cytotoxic and genotoxic activity toward all tested cell types. In conclusion, our results suggest that **MM129**, **MM130**, and **MM131** exhibit high cytotoxic and genotoxic potential in vitro, especially towards the colorectal cancer cell line HCT 116. However, further investigations and analyses are required for their future implementation in the field of medicine.

## 1. Introduction

According to recent data, in 2020 nearly 10 million people died due to cancer, which is now the leading cause of death worldwide. Lack of physical activity and high body mass index, as well as tobacco and alcohol use, are the main factors associated with an elevated risk of cancer. They are considered to be the reason for approximately one-third of cancer deaths. Breast, lung, colon and rectum, prostate, skin (non-melanoma), stomach, and cervical cancers are the most common types of malignancies [1,2]. Despite the remarkable progress in the development of different methods for the treatment of cancer, including surgery, radiation therapy, endocrine therapy, gene therapy, and immunotherapy, chemotherapy remains the first line of cancer treatment. This is the reason why searching for novel effective antineoplastic agents is one of the most broadly investigated topics worldwide [3,4,5,6,7,8].

**Scheme 1:**
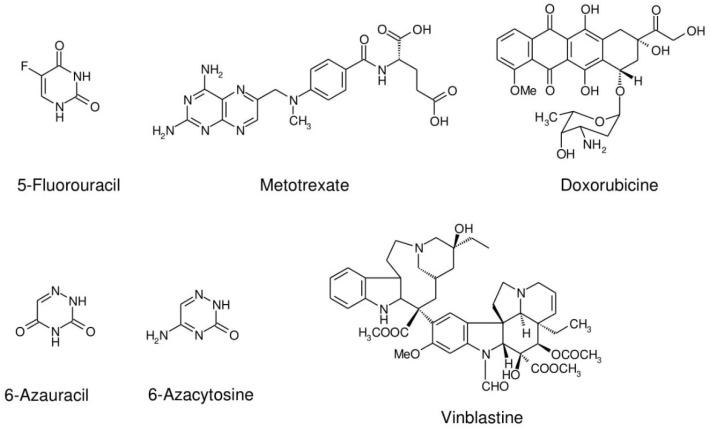
Anticancer drugs used in medicine.

Several commonly used anticancer drugs in medicine are heterocyclic compounds, such as 5-fluorouracil, methotrexate, doxorubicin, vinblastine, etc. (Scheme 1). However, heterocyclic core and/or substituent structures are still the base for the design and synthesis of new biologically active molecules. Antimetabolite drugs with a chemical structure similar to nitrogenous bases in DNA/RNA are considered as one of the main groups of antineoplastic agents. These anticancer drugs not only disturb DNA/RNA biosynthetic pathways that are key for maintaining cell growth, but are also responsible for the formation of DNA strand breaks. The incorporation of purine/pyrimidine analogs into DNA or inhibition of the activity of some enzymes, such as ribonucleotide reductase, dihydrofolate reductase, and DNA polymerase may generate strand breaks [9,10]. Numerous nucleosides that contain interchanged carbon and nitrogen atoms in their base moieties have presented significant potential as antimetabolite agents. 6-Aza compounds, such as 6-azacytosine and 6-azuracil, are well-known examples of the nucleic acids components analogs [11,12]. The group of aza-compounds also includes monocyclic 1,2,4-triazine derivatives. These monocyclic 1,2,4-triazine-based compounds display various biological activities, including anticancer, antimicrobial, antifungal, anti-inflammatory, antimalarial, and antiviral properties [13,14]. Due to the fact that 1,2,4-triazines fused with five-membered heterocycles are considered as bioisosteric with a purine core, these compounds have attracted considerable attention in the field of medical chemistry. The group of isosteric purine analogs also includes derivatives of the little-known pyrazolo[4,3-*e*][1,2,4]triazine ring system [15]. They exhibit various biological properties, including antibacterial and anticancer activities [16,17,18]. It has been revealed that some synthetic pyrazolo[4,3-*e*][1,2,4]triazine sulfonamides display inhibitory activity towards urease [19], tyrosinase [15,16], carbonic anhydrase (CA) [20,21], and protein kinases (Bcr-Abl and CDK2) [22].

There are 538 protein kinases encoded by the human genome [23,24]. Amplifications or mutations of the cyclin-dependent kinases (CDKs), the main group of cell cycle regulatory proteins, are commonly observed in malignant cells [25]. The ability to inhibit the kinase-signaling pathways has particular importance due to the possibility of more personalized tumor treatment with decreased risk of cytotoxicity in normal cells [26]. A fusion gene *BCR-ABL*, which is frequently found in patients with chronic myeloid leukemia (CML), is responsible for enhanced resistance to apoptosis and increased proliferation of myeloid cells, as well as their adhesion properties [27,28,29,30,31]. Interestingly, blocking only a single ATP-binding site of the particular protein kinase prevents phosphorylation in *BCR-ABL*-expressing cells, resulting in their apoptosis or growth inhibition [29]. Among many carbonic anhydrase (CA) isoforms, there are two whose overexpression is correlated with tumor development and progression—hCA IX and hCA XII. Results of Mojzych et al. [20,21] have shown that two of investigated pyrazolo[4,3-*e*][1,2,4]triazine derivatives significantly inhibited cancer-associated isoenzymes hCA IX and XII, and were ineffective toward non-tumor isoenzymes hCA I and II.

Furthermore, some of our previously synthesized pyrazolo[4,3-*e*][1,2,4]triazine derivatives with a tetrazole or triazole ring exhibited strong antineoplastic properties toward several cancer cell lines [32] (colorectal adenocarcinoma (Colo205)—IC_50_ 4–91 μM; breast cancer (MCF-7)—IC_50_ 50–90 μM; prostate cancer (PC-3)—IC_50_ 25–98 μM; non-small-cell lung cancer (H460) IC_50_ 25–86 μM). These results encouraged us to design a novel group of tricyclic derivatives, i.e., pyrazolo[4,3-*e*]tetrazolo[1,5-*b*][1,2,4]triazine with sulfonamidophenyl substituent. Sulfonamide moiety is a functional group present in various drugs, including antineoplastic agents. Moreover, aryl/heteroaryl compounds can disturb microtubule synthesis and stop the cell cycle in the G1 phase, as well as block tumor angiogenesis [33]. Bearing in mind the above literature reports, in this paper, we present the cytotoxicity and genotoxicity of the three sulfonamide derivatives of the new pyrazolo[4,3-*e*]tetrazolo[1,5-*b*][1,2,4]triazine ring system in vitro: **MM129**, **MM130**, and **MM131**. Importantly, anticancer properties of tested compounds against prostate cancer (PC-3), cervical cancer (HeLa), colorectal carcinoma (HCT 116), and pancreatic adenocarcinoma (BxPC-3) have not been examined so far.

## 2. Results

### 2.1. Synthesis of Novel 1,2,4-Triazine Derivatives

**Scheme 2:**
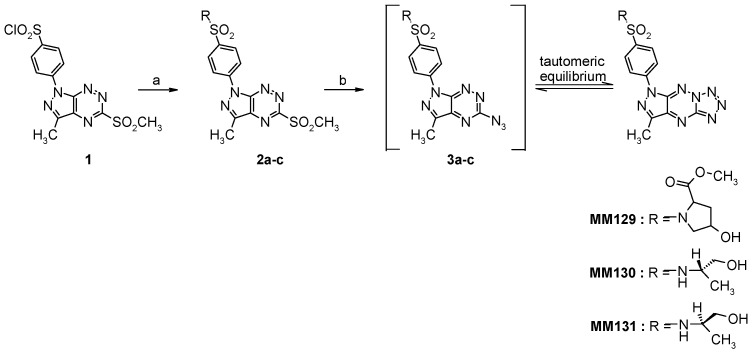
Reagents and conditions: (**a**) (*R*)- or (*S*)-2-aminopropanol or *cis*-4-hydroxy-l-proline methyl ester hydrochloride, MeCN, rt, overnight; (**b**) NaN_3_, EtOH, reflux, 18 h.

The target tricyclic sulfonamides (**MM129**, **MM130**, and **MM131**) were obtained according to our general multi-step procedure described in the literature (Scheme 2) [34]. Briefly, the corresponding chlorosulfone derivative **1**, achieved in multi-step synthesis, is the pivotal starting material for the synthesis of tricyclic sulfonamides [34,35]. The general character of the developed method synthesis enables the functionalization of the heterocyclic core by replacing the chlorine atom in the chlorosulfone group with any amine derivative.

The reaction of **1** with the appropriate amine derivatives yields suitable sulfonamides **2a**–**c** with a good leaving group (-SO_2_CH_3_) that undergoes nucleophilic displacement upon reaction with sodium azide and leads in the first step to the formation of intermediates **3a**–**c** that, under reaction conditions, can undergo intramolecular cyclization to afford final tricyclic sulfonamide derivatives **MM129**, **MM130 and MM131** [34]. All intermediates and final derivatives were characterized using the ^1^H- and ^13^C-NMR, and HRMS methods together with elemental analysis. The obtained spectroscopic data for **1, 2a**, **2c**, **MM129**, and **MM131** were consistent with the literature and fully confirmed the structure of these compounds (Supplementary Materials Figures S1–S19).

### 2.2. Cytotoxicity—MTT Assay

**Figure 1:**
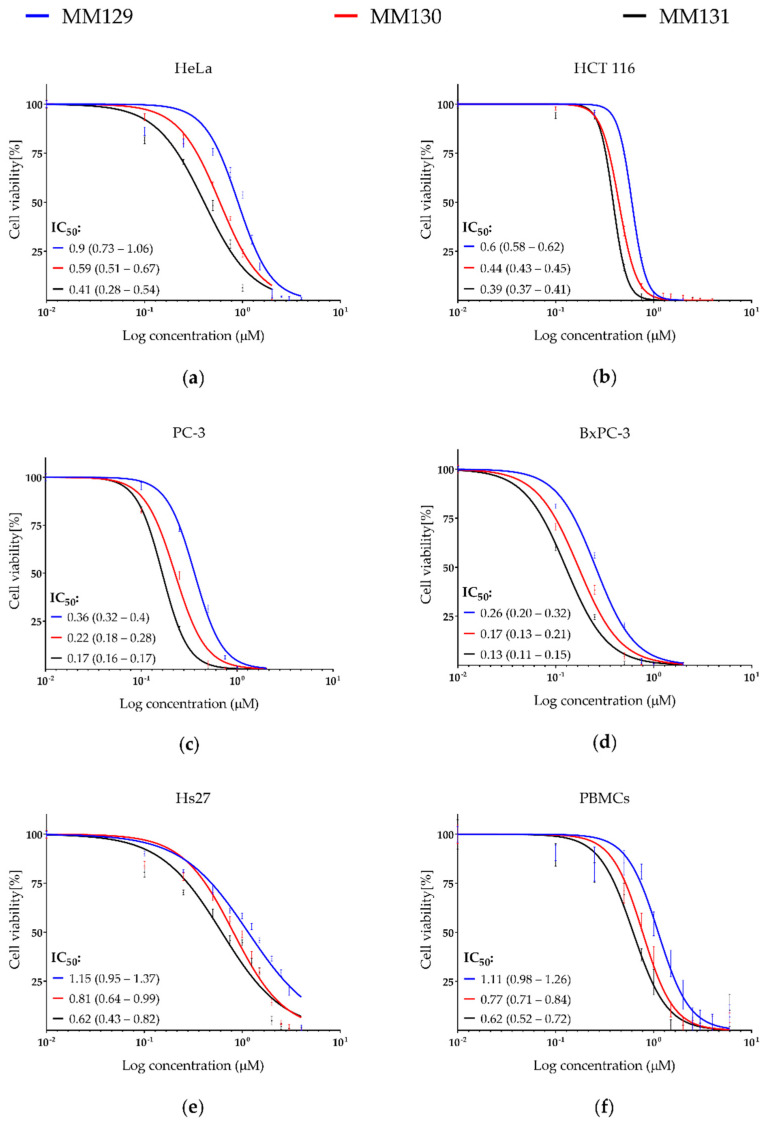
Evaluation of cell viability (%) of cancer cell lines: (**a**) HeLa; (**b**) HCT 116; (**c**) PC-3; (**d**) BxPC-3 and normal cells: (**e**) Hs27 and (**f**) PBMCs treated with **MM129**, **MM130,** and **MM131**. The survival curves ± SEM were presented for each tested compound. IC_50_ values and 95% confidence intervals (parentheses) are given in μM.

The results obtained from the MTT assay (72 h incubation period) show that new tricyclic derivatives of the pyrazolo[4,3-*e*][1,2,4]triazine are more cytotoxic toward tested cancer cell lines in comparison to human peripheral blood mononuclear cells (PBMCs) and the fibroblast Hs27 (human foreskin) (Figure 1; examined compounds have shown 1.2–4.9-times higher cytotoxicity in cancer cell lines compared to the normal cells). Among tested pyrazolo[4,3-*e*]tetrazolo[1,5-*b*][1,2,4]triazine sulfonamides **MM129** exhibited the lowest cytotoxic activity, while **MM131** presented the highest one. The reduction in cell viability corresponded with the increasing concentration of examined compounds. The sensitivity of the cells from the lowest to the highest is presented as follows: Hs27, PBMCs, HeLa, HCT 116, PC-3, and BxPC-3 (Figure 1).

### 2.3. Genotoxicity

#### 2.3.1. Comet Assay

##### Alkaline Version (pH > 13)

**Figure 2:**
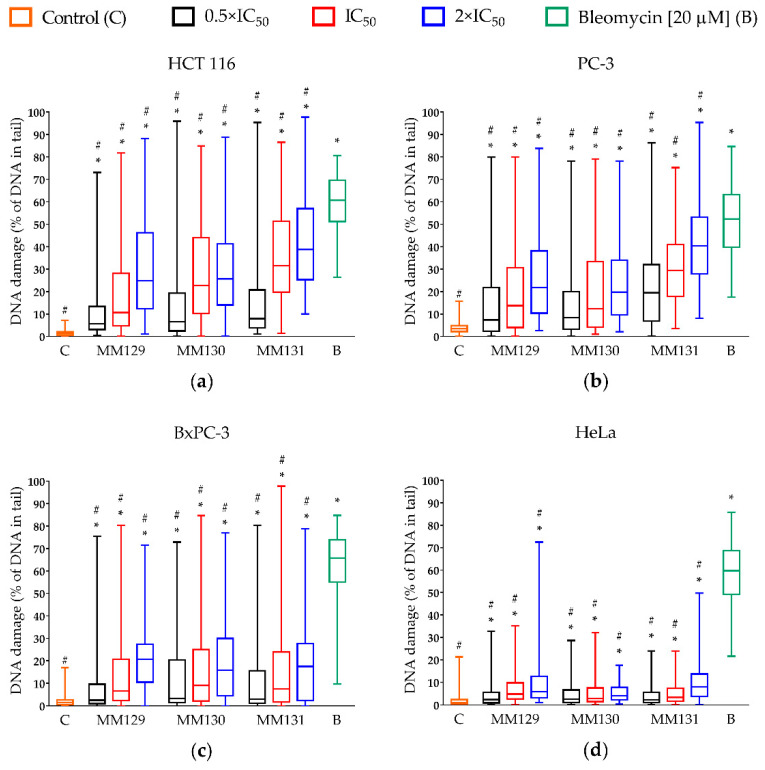
Evaluation of DNA damage (SSBs, DSBs, and alkali-labile sites) in human cancer cell lines: (**a**) HCT 116; (**b**) PC-3; (**c**) BxPC-3; (**d**) HeLa treated with **MM129**, **MM130**, and **MM131** in the concentrations of 0.5, 1, and 2 × IC_50_ for 24 h using the alkaline version of the comet assay. The applied concentrations of investigated compounds were at least 10 times lower than the concentration of bleomycin (positive control) used in the experiment. DNA damage was shown as a median of the percentage of DNA in comet tail with interquartile range and minimal and maximal values. Significant differences (*p* < 0.0001) compared with negative (*) and positive (#) control.

The genotoxic potential of examined derivatives was assessed only in cancer cells. Figure 2 represents the level of DNA damage (SSBs, DSBs, alkali labile sites) induced by **MM129**, **MM130**, **MM131**, and bleomycin following 24 h incubation of the cells and measured as a percentage of DNA in comet tail. The results show that each of the examined pyrazolo[4,3-*e*]tetrazolo[1,5-*b*][1,2,4]triazine sulfonamides significantly (*p* < 0.0001) induced DNA damage, even at their lowest concentrations (0.5 × IC_50_), compared to untreated cells, in all cancer cell lines in a concentration–response manner.

**Figure 3:**
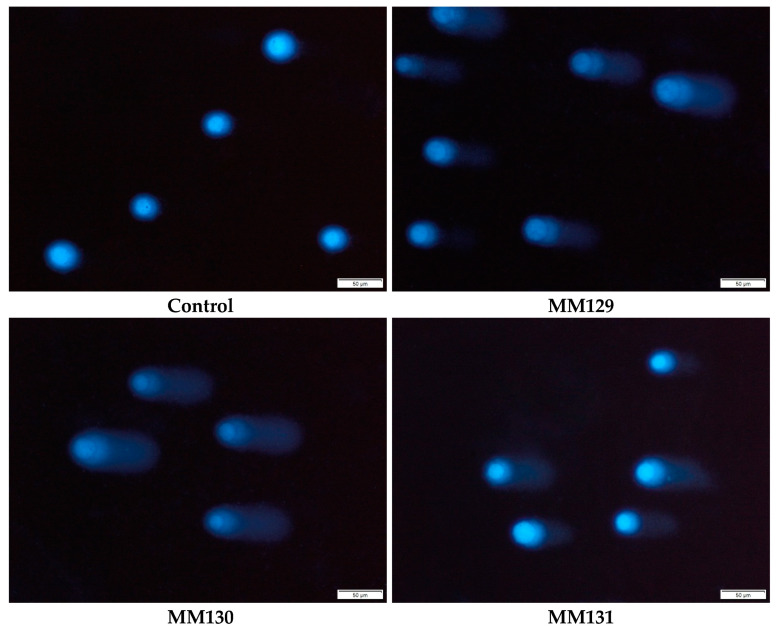
Exemplary images of the comets obtained in the alkaline version of the comet assay after incubation of PC-3 cells with **MM129**, **MM130**, and **MM131** in the concentrations equal to their 2 × IC_50_ values. The scale bar is 50 μm.

Differences in response of the cells to genotoxic activity of investigated compounds were noticed. From all tested compounds, **MM131** exhibited the strongest genotoxic activity against PC-3 and HCT 116 cells (19.5–40.3% and 8–38.8% of DNA in comet tail, respectively). The moderate effect of investigated compounds with no significant differences (*p* > 0.05) between their genotoxic activity was observed in the BxPC-3 and the HeLa cell line (2.5–20.7% and 2.3–8.1% of DNA in comet tail, respectively). Figure 3 demonstrates the representative photos of comets from this experiment.

##### Neutral Version (pH 9.0)

Table 1 shows the comparison of DNA damage level in both versions of the comet assay measured as a percentage of DNA in the comet tail, after 24 h incubation with tested compounds. Among the investigated cells, HCT 116 showed the highest level of DSBs induced by examined pyrazolo[4,3-*e*]tetrazolo[1,5-*b*][1,2,4]triazine sulfonamides (15.8–30.6% of DNA in the tail), while DNA damage levels in BxPC-3 and PC-3 cell lines were in the range of 7.1–10% and 1.5–7.4% of DNA in the comet tail, respectively.

**Figure 4:**
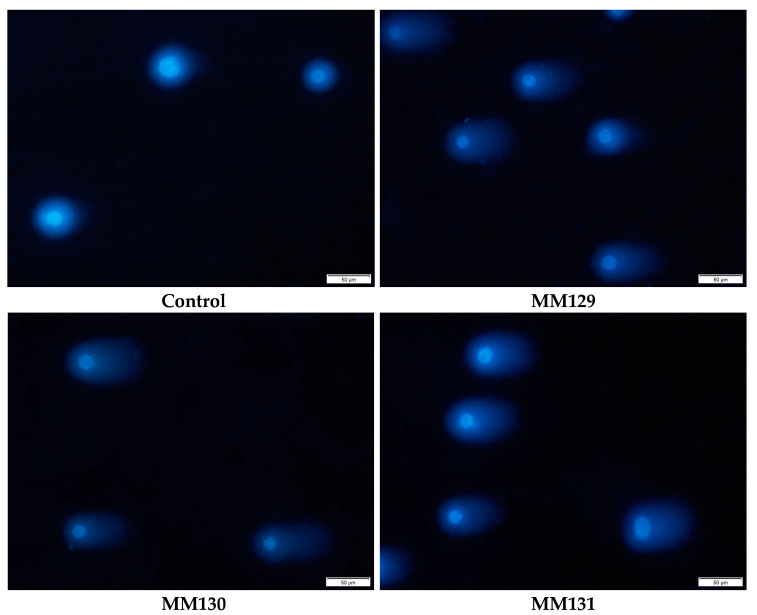
Representative images of comets acquired in the neutral version of the comet assay after incubation of HCT 116 cells with **MM129**, **MM130**, and **MM131** in the concentrations equal to their 2 × IC_50_ values. The scale bar is 50 μm.

The HeLa cell line was omitted in the neutral comet assay, due to its low response to the examined compounds in comparison to other cell lines in the alkaline version of the comet assay. The compounds that presented the strongest genotoxic activity in the HCT 116 and PC-3 cell lines were **MM130** and **MM129**, respectively. There was no significant difference (*p* > 0.05) between the genotoxicity of tested sulfonamides in BxPC-3 cells (Table 1). Figure 4 demonstrates the representative photos of comets from this experiment.

#### 2.3.2. Immunocytochemical Detection of γ-H2AX

**Figure 5:**
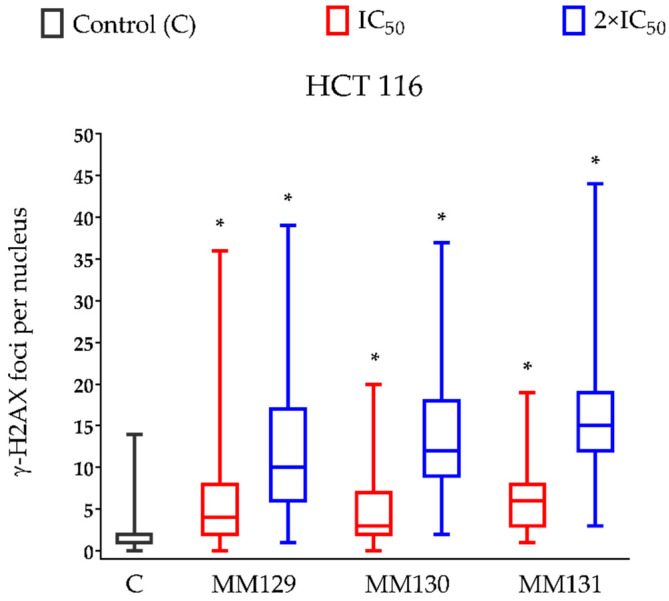
The presence of the γ-H2AX foci of DSBs per nucleus in HCT 116 cells treated with **MM129**, **MM130**, and **MM131** for 24 h. The data are shown as a median of the number of γ-H2AX foci with interquartile range and minimal and maximal values. Significant differences (*p* < 0.0001) compared with negative control (*).

**Figure 6:**
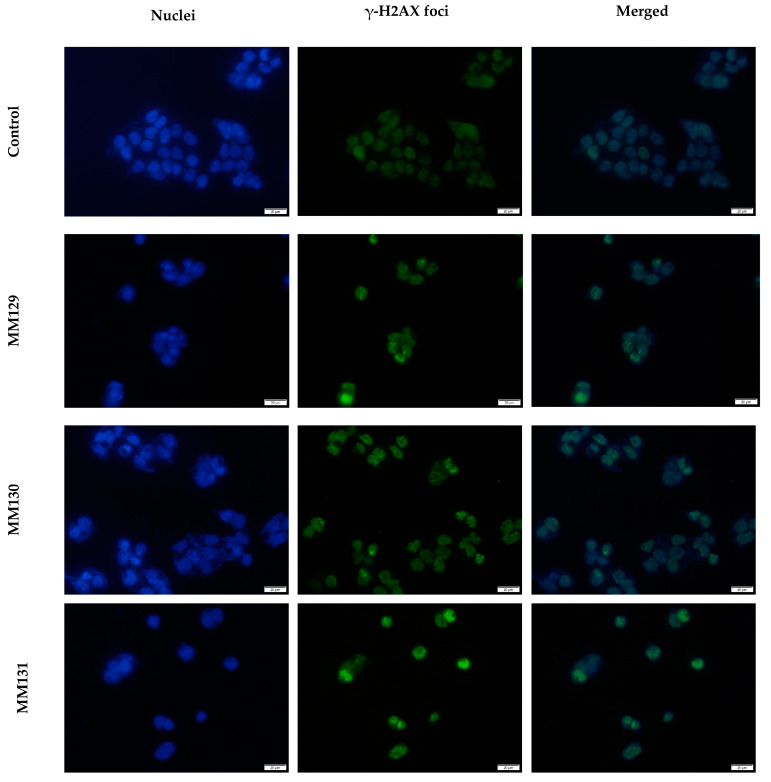
Exemplary images of γ-H2AX foci in HCT 116 cells after 24 h treatment with MM compounds in the concentrations equal to their 2 × IC_50_ values; nuclear DNA stained with DAPI (blue); γ-H2AX foci (green). The scale bar is 20 μm.

The γ-H2AX staining was employed to confirm the genotoxic properties of examined compounds presented in the neutral comet assay. The HCT 116 cell line was the most sensitive to the DSBs induction (Table 1) and was therefore selected for this analysis. Incubation of HCT 116 cells for 24 h with examined compounds caused an increase in the levels of DNA damage, measured as a median of the number of γ-H2AX foci per nucleus in these cells. Significance differences (*p* < 0.0001) between compound-treated and control cells were noticed. The concentration–response relationship was observed for each examined compound. From all investigated derivatives of pyrazolo[4,3-*e*]tetrazolo[1,5-*b*][1,2,4]triazine, **MM131** exhibited the strongest genotoxic potential (median of the number of γ-H2AX foci per nucleus for the highest concentration used in the study was 15) (Figure 5). Figure 6 demonstrates the representative pictures of γ-H2AX foci in HCT 116 cells from this experiment.

## 3. Discussion

Antineoplastic properties of several MM compounds, including MM124, MM137 [34], **MM129** [35,36], and **MM131** [37] with promising results have been examined so far. The mechanistic study showed that down-regulation of PD-L1 (programmed death-ligand 1) expression, which plays a fundamental role in the immunological self-tolerance, was correlated with the use of **MM129**. Moreover, data indicate that **MM129** can inhibit intracellular molecules associated with cell cycle arrest and tumorigenesis promotion, such as CDK2, mTOR, or AKT [36]. In the present study, we showed for the first time cytotoxic and genotoxic potential of **MM129**, **MM130**, and **MM131** towards HCT 116, HeLa, PC-3, and BxPC-3 cancer cell lines.

Data obtained by Gornowicz et al. [34] demonstrated high cytotoxicity of MM124 and MM137 against colorectal cancer (CRC) cell lines HT-29 and DLD-1 (IC_50_ values in the range of 0.16–1.54 µM). In addition, the results presented by Hermanowicz et al. [35] and Gornowicz et al. [37] have shown cytotoxic activity of **MM129** and **MM131** towards the same CRC cell lines (IC_50_ values in the range of 3.1–3.9 µM). Importantly, in all cases, obtained IC_50_ values were much lower than those for the reference drugs 5-fluorouracil and/or roscovitine (RSC) [34,35,37]. Our results of the MTT assay confirmed cytotoxic activity of previously mentioned triazine derivatives **MM129** and **MM131** towards another CRC cell line (HCT 116), as well as other cancer cell lines, including HeLa, PC-3, and BxPC-3, in a similar concentration range (Figure 1; IC_50_ = 0.13–0.9 μM). In this research, the cytotoxicity of **MM130** was demonstrated for the first time. **MM130** presented moderate cytotoxic potential in comparison to other MM compounds in all studied cancer cell lines. Despite the high cytotoxic potential of the tested compounds towards the CRC cell line HCT 116 (IC_50_ in the range of 0.39–0.6 µM), the tested derivatives of pyrazolo[4,3-*e*]tetrazolo[1,5-*b*][1,2,4]triazine showed even more profound cytotoxic activity against PC-3 and BxPC-3 cell lines (IC_50_ in the range of 0.17–0.36 µM and 0.13–0.26 µM, respectively). Importantly, the higher resistance of normal PBMCs and Hs27 cells compared to the cancer cell lines toward the tested tricycle triazine derivatives (Figure 1; cancer/normal cells IC_50_ ratio showed that MM compounds were 1.2–4.9-times more cytotoxic in cancer cell lines compared to normal cells) was observed (Figure 1).

Based on the criteria demonstrated by National Cancer Institute (NCI), the cytotoxicity of a compound can be classified as high, moderate, and weak (IC_50_ < 20 µg/mL, 21–200 µg/mL, or in the range of 201–500 µg/mL, respectively). All investigated sulfonamides are classified as compounds with high cytotoxicity [38]. In addition, the examined MM compounds exhibit significantly stronger cytotoxicity in comparison to various currently used chemotherapeutics, such as 5-fluorouracil (IC_50_–96.1 µM for BxPC-3 and 3.2 µM for HCT 116 [39,40]), cisplatin (IC_50_–9 µM for PC-3 and HCT 116 and 12.3 µM for HeLa [41,42]), oxaliplatin (IC_50_–72 µM for HCT 116 and 100 µM for PC-3 [42]), or irinotecan (IC_50_–2.1 µM for PC-3 and 2.6 µM for HCT 116) [40,43]. The limitation of doses of antineoplastic agents seems to be crucial for the reduction in side effects that occur during chemotherapy treatment. Further investigations of the anticancer properties of **MM129**, **MM130**, and **MM131** are required; however, their high cytotoxicity indicates the potential implementation of these compounds in the field of cancer treatment.

Inhibition of cell proliferation caused by DNA SSBs and DSBs formation is one of the main mechanisms of the action of various antineoplastic drugs. The creation of adducts and/or interactions of free radicals with the double helix of DNA plays a fundamental role in DNA degradation. DNA DSBs, due to their extreme difficulty to repair, are the most serious threat to the cell. Unrepaired DSBs may promote cell death, while misrepaired ones can be a potential factor for further neoplastic progression [44,45,46]. DNA DSBs induced through factors such as chemotherapeutic agents or ionizing radiation result in phosphorylation of H2AX on its residue serine 139 via kinases, such as ataxia telangiectasia mutated (ATM), DNA-dependent, and ATM-RAD-3-related protein kinase [47,48,49,50,51,52]. The phosphorylation of H2AX leads to recruitment of MRN (hRAD50/hMRE11/NBS1) and mediator of the DNA damage checkpoint (MDC1) repair complex, which enhances the ATM activation, and subsequently results in phosphorylation even more of H2AX histone proteins surrounding the DSB sites. MDC1 works as a platform for other DDR components that enhance the conveyed signal and activate DNA repair pathways through nonhomologous end-joining or homologous recombination via recruitment of 53BP1 or BRCA1 proteins, respectively. The studies have shown that the number of DNA DSBs in cells directly correlates with the number of γ-H2AX foci, which are considered as a well-known marker for DSBs [53].

The high genotoxicity of investigated compounds towards HCT 116 cells, especially the high level of DNA DSBs measured via the neutral comet assay (Table 1) and by the immunocytochemical detection of γ-H2AX (Figure 5), is worth noting. Despite strong genotoxic properties of **MM129**, two other MM compounds, **MM130** and **MM131,** exhibited similar or even higher genotoxic potential toward HCT 116 cells, measured in the alkaline, as well as in the neutral version of the comet assay (Table 1). Moreover, the median number of γ-H2AX foci in HCT 116 cells after 24 h incubation with **MM130** and **MM131** was higher when compared to **MM129** (Figure 5). The results of the alkaline version of the comet assay strongly indicate higher genotoxicity of **MM131** when compared to **MM130** at the concentrations equal to their 2 × IC_50_ values (Figure 2; 38.8% vs. 25.6% of the DNA in the tail, respectively). Differences between the genotoxic activity of **MM130** and **MM131** in the neutral comet assay and γ-H2AX staining are slightly more subtle. Immunocytochemical detection of γ-H2AX showed the strongest genotoxic potential of **MM131** (Figure 5; median of 12 vs. 15 foci per nucleus for **MM130** and **MM131** at their highest concentrations used, respectively). However, the results of the neutral comet assay revealed stronger genotoxicity of **MM130**, when compared to **MM131** for both IC_50_ and 2 × IC_50_ values (Table 1).

The results obtained from the alkaline version of the comet assay demonstrated high genotoxic potential of tested compounds not only towards HCT 116 cells, but also against PC-3 and BxPC-3 cell lines (Figure 2). Surprisingly, HeLa was much more resistant to genotoxic activity of the investigated tricycle triazine derivatives (Figure 2; 4.1–8.1% of the DNA in the comet tail for higher concentrations of tested compounds). To find an explanation of this dissimilarity, further investigation of **MM129**, **MM130**, and **MM131** against the HeLa cell line is required. Interestingly, **MM131** exhibited the strongest genotoxic activity the among tested derivatives of pyrazolo[4,3-*e*]tetrazolo[1,5-*b*][1,2,4]triazine on the PC-3 cells (Figure 2; 19.5–40.3% of the DNA in the comet tail for **MM131** vs. 7.4–21.8% of the DNA in the comet tail for **MM129** and **MM130**). There were no significant differences (*p* > 0.05) observed between **MM129** and **MM130** genotoxicity for the PC-3 cell line. Furthermore, similar genotoxicity, with no significant difference within examined MM compounds (*p* > 0.05), was noticed in the case of the BxPC-3 and HeLa cell lines (Figure 2). The only cell line that demonstrated comparable DNA damage in both versions of the comet assay was HCT 116, suggesting that DSBs are the major DNA damage induced in these cells (Table 1; 38.8% vs. 30.6% of DNA in the comet tail for the higher used concentrations). Nevertheless, even if the level of the DSBs determined in the BxPC-3 and PC-3 cells after the MM compounds’ treatment was relatively low, unrepaired SSBs can lead to DSB formation and initiate cell death through the apoptosis.

High antineoplastic properties of **MM129** were confirmed not only by an in vitro model but also via an in vivo model. During the two weeks of **MM129** treatment, a significant reduction in tumor mass and volume in the mouse model of xenotransplantation (mice challenged with HT-29 and DLD-1 cancer cells) was observed [54]. Furthermore, in the previous study of Hermanowicz et al. [31], the inhibition of tumor development in the zebrafish embryo xenograft model was correlated with **MM129** activity. Our results on another CRC cell line are in agreement with these data. In the present paper, the HCT 116 cell line was the most sensitive to DNA DSBs formation induced by **MM129** among tested cancer cells (Table 1). Immunocytochemical detection of γ-H2AX confirmed the results obtained in the neutral comet assay, showing the highly genotoxic potential of **MM129** against HCT 116 cells (Figure 5). We speculate that the efficiency of anticancer activity of **MM129** against colon cancer in Cby.Cg-Foxn1nu/cmdb mice, as well as in the zebrafish embryo xenograft model, may be correlated with strong genotoxic activity of **MM129**.

Higher cytotoxic and genotoxic properties of **MM130** and **MM131** vs. **MM129** are due to differences in one moiety between those compounds—*cis*-4-hydroxy-l-proline methyl ester hydrochloride present in **MM129** vs. 2-aminopropanol presents as stereoisomers in the other two compounds. However, to find a proper molecular explanation for these differences in anticancer activity between the remaining two compounds, molecular target identification and in silico studies of examined substances are required. Nevertheless, despite the same moiety in **MM130** and **MM131**, in our study **MM131** inhibited stronger cytotoxic and genotoxic potential than **MM130** (Figure 1, Figure 2 and Figure 5). Data show that differences between stereoisomers are associated with their pharmacokinetic properties, such as their absorption, bioavailability, distribution, and metabolism, as well as their pharmacological potency and activity. Because of these differences, some of them can be useful in some fields of medicine, while the others may be extremely dangerous (*S*-Thalidomide, *S*-Naproxen) or biologically inactive (*R*-ibuprofen) [55]. We suggest that the differences in cytotoxicity, as well as in genotoxicity of **MM130** and **MM131** (Table 1 and Figure 1, Figure 2 and Figure 6) may result from their stereoisomerism. To confirm our speculations, further investigations of the biological activities, as well as the pharmacokinetic and pharmacodynamics properties of both compounds, are required.

## 4. Materials and Methods

### 4.1. Synthesis

#### 4.1.1. General

Melting points were determined on a Mel-Temp apparatus and were uncorrected. ^1^H- and ^13^C-NMR spectra were recorded on a Varian spectrometer (400 MHz for ^1^H and 100 MHz for ^13^C). The chemical shift values were expressed in ppm (part per million) with tetramethylsilane (TMS) as an internal reference. The relative integrals of peak areas agreed with those expected for the assigned structures. The molecular weight of the final compounds was assessed by electrospray ionization mass spectrometry (ESI/MS) on Agilent Technologies 6538 UHD Accurate Mass Q-TOF LC/MS. Elemental analyses were within ±0.4% of the calculated values. For the preparation and spectroscopic data of the compounds **2a, 2c, MM129,** and **MM131**, see the literature [34,35].

#### 4.1.2. Preparation of N-(S)-(1-hydroxy-propan-2-yl)-4-(3-methyl-5-methylsulfonyl-1H-pyrazolo[4,3-*e*][1,2,4]triazin-1-yl)benzenesulfonamide (**2b**)

Chlorosulfonyl derivative **1** [34] (194 mg, 0.5 mmol) was dissolved in anhydrous acetonitrile (10 mL) and (*S*)-2-aminopropanol (131 mg, 1.75 mmol) was added and stirred overnight at room temperature. Then, the reaction mixture was concentrated in vacuo to afford the crude product **2b**. The residue was purified on silica gel using a mixture of CH_2_Cl_2_:EtOH (25:1) as eluent to give the final sulfonamide as a yellow solid.

Yield 96%. Melting point: 215–220 °C; ^1^H-NMR (methanol) δ: 1.03 (d, 3H, *J* = 6.4 Hz), 2.85 (s, 3H), 3.33–3.38 (m, 2H), 3.43–3.50 (m, 1H), 3.57 (s, 3H), 4.57 (bs, 1H, NH), 8.13 (d, 2H, *J* = 8.8 Hz), 8.65 (d, 2H, *J* = 8.8 Hz); ^13^C-NMR (methanol) δ: 11.12, 17.98, 41.04, 52.55, 66.90, 121.36, 129.62, 139.31, 141.45, 147.73, 149.90, 162.88, 210.13. HRMS (ESI, *m/z*) Calcd for C_15_H_19_N_6_O_5_S_2_ [M + H] 427.1322. Found [M + H] 427.1320. Anal. Calcd for C_15_H_18_N_6_O_5_S_2_: C, 42.25; H, 4.25; N, 19.71. Found: C, 42.50; H, 4.47; N, 19.54.

#### 4.1.3. Synthesis of *N*-(*S*)-(1-hydroxy-propan-2-yl)-4-[7-methyl-5*H*-pyrazolo[4,3-*e*]tetrazolo[1,5-*b*][1,2,4]-triazin-5-yl)]benzenesulfonamide (**MM130**)

To a solution of compound **2b** (140 mg, 0.33 mmol) in anhydrous ethanol (25 mL), sodium azide (21 mg, 0.33 mmole) was added. The reaction mixture was refluxed until the substrate disappeared (control TLC). Then, the solvent was evaporated, and the crude product was purified using column chromatography and CH_2_Cl_2_: MeOH (50:1) mixture as eluent to give the final sulfonamide as a yellow solid.

Yield 91%. Melting point: 197–202 °C; ^1^H-NMR (methanol) δ: 1.01 (d, 3H, *J* = 6.4 Hz), 2.85 (s, 3H), 3.30–3.36 (m, 2H), 3.43–3.48 (q, 1H, *J* = 5.6 Hz), 4.58 (bs, 1H, NH), 8.10 (d, 2H, *J* = 8.8 Hz), 8.45 (d, 2H, *J* = 8.8 Hz); ^13^C-NMR (methanol) δ: 11.18, 17.96, 52.51, 66.87, 120.21, 129.66, 140.65, 141.93, 143.32, 148.29, 148.85, 149.32. HRMS (ESI, *m/z*) Calcd for C_14_H_16_N_9_O_3_S [M + H] 390.13220. Found [M + H] 390.13210. Anal. Calcd for C_14_H_15_N_9_O_3_S: C, 43.18; H, 3.88; N, 32.37. Found: C, 43.40; H, 4.02; N, 32.15.

### 4.2. Chemicals

Trypsin/EDTA and media used for cell culture (DMEM, DMEM-F12, IMDM, and RPMI 1640), as well as gradient cell separation medium used for PBMCs isolation (Lymphosep), were supplied by Biowest (Nuaillé, France). Normal-melting-point (NMP) agarose, low-melting-point (LMP) agarose, Triton X-100, 3-(4,5-dimethylthiazol-2-yl)-2,3-diphenyltetrazolium bromide (MTT), dimethyl sulfoxide (DMSO), 4,6-diamidino-2-phenylindole (DAPI), penicillin–streptomycin solution stabilized, fetal bovine serum (FBS), phytohaemagglutinin (PHA), phosphate-buffered saline (PBS), and *N*,*N*-Dimethylformamide (DMF) were purchased from Sigma-Aldrich (St Louis, MO, USA). Microplates, as well as 96-well and 12-well plates, were supplied by Thermo Fisher Scientific (Waltham, MA, USA). Other chemicals used in the experiments: fluoromount-G (Invitrogen, Carlsbad, CA, USA); bleomycin (TCI); SDS (ROTH); paraformaldehyde (Polysciences, Inc; 400 Valley Rd, Warrington, PA 18976, USA); goat serum (Abcam, Cambridge, UK); the primary antibody, anti-gamma-H2AX (phospho-Ser139) (Abcam, Cambridge, UK); the secondary Goat anti-Mouse IgG Cross-adsorbed antibody, Alexa Fluor 488 p (Invitrogen, Carlsbad, CA, USA).

### 4.3. Cell Culture

All tested adherent human cell lines, including Hs27, PC-3, HeLa, HCT 116, and BxPC-3, were provided by American Type Culture Collection (ATCC^®^, Rockville, Manassas, VA, USA). PC-3 and HeLa cells were cultivated in DMEM-F12 and IMDM media, respectively, while RPMI 1640 medium was used for HCT 116 and BxPC-3 cells. DMEM was used for the Hs27 cell line. All media were supplemented with 10% (*v*/*v*) FBS and 1% (*v*/*v*) penicillin–streptomycin solution. Each of the adherent human tumor cell lines was maintained in a humidified incubator at 37 °C in a 5% CO_2_ atmosphere and was regularly scanned for mycoplasma contamination. In order to maintain the proper amount of culture cells, subculture was performed by their routine passaging at 90% confluence three times a week using 0.025% trypsin/EDTA. Furthermore, to provide the cells with stable growth conditions, the culture medium was changed every 48 h.

Human PBMCs were isolated from the leucocyte buffy coat obtained from the blood of healthy and nonsmoking volunteers (aged 18–65). Leukocyte buffy coats were collected from Blood Bank in Lodz, Poland. The use of the leukocyte buffy coat in the investigation of the effect of novel tricyclic pyrazolo[4,3-*e*][1,2,4]triazine derivatives on human PBMCs was approved by the Bioethics Committee for Scientific Investigation, University of Lodz (agreement no. 8/KBBN-UŁ/I//2019). Isolation of PBMCs was performed by density gradient of Lymphosep (25 min, 1400 rpm, RT). PBMCs were cultured in RPMI-1640 medium, supplemented with 1% (*v*/*v*) penicillin–streptomycin solution, 1.5% PHA and 10% (*v*/*v*) FBS.

Each experiment on adherent human cell lines or human PBMCs was conducted on cells from three independent passages or cells obtained from the blood of three independent leukocyte buffy coats, respectively.

### 4.4. Cytotoxicity—MTT Assay

In order to characterize the effect of tested compounds on cell viability, the MTT test was used [56]. MTT microplate assay enables evaluation of cell viability via measuring cell metabolic activity represented as an ability to incorporate and reduce yellow tetrazolium salt to the violet-blue formazan compounds via the activity of mitochondrial succinate dehydrogenase enzyme. The number of metabolically active cells positively correlates with the amount of reduced tetrazolium salt. The absorbance of formazan solution obtained after solubilizing formazan crystals in an organic solvent, e.g., DMSO, can be measured spectrophotometrically at 570 nm [57].

The suspension of 8–10 × 10^3^ (adherent cells) and 8 × 10^4^ (PBMCs) cells in 200 µL and 100 µL medium, respectively, was transferred to each well of 96-well microplate. Plates were incubated for 24 h in controlled conditions (5% CO_2_; 37 °C) to ensure cell growth. Subsequently, the medium was replaced, and the cells were exposed to tested compounds in a wide range of concentrations (0.1–6 µM). Before investigated compounds were diluted in a culture medium, they had been diluted in DMSO in the final concentration of <0.5% *v*/*v*, which were not toxic to the cells [58,59]. The experimental design included blanks (wells without cells) as well as non-treated control cells. Following the 72 h incubation period, a volume of 20 µL of fresh tetrazolium salt solution (5 mg/mL in PBS) was added per well. In the case of adherent cancer cells, after 3 h of incubation (conditions as previously), the solutions were replaced by 100 µL of DMSO to dissolve formazan complexes. In contrast, the solutions in plates containing PBMCs were not removed and a 100 µL mixture of 50% DMF and 20% SDS was added to each well for the next 24 h. Absorbance was measured via spectrophotometer (microplate reader Power Wave XS BioTek Instruments, Inc., Winooski, VT, USA) at 570 nm. To determine the IC_50_ values (concentration of tested inhibitor, which decreases the cell metabolic rate by 50%, expressed as the reduction in absorbance level in treated cells in comparison to untreated cells by half) of studied compounds, the concentration–response analysis was conducted in a GraphPad Prism 8.0 software system (GraphPad Prism Software Inc., San Diego, CA, USA).

### 4.5. Genotoxicity

#### 4.5.1. Comet Assay

The alkaline version (pH > 13), as well as the neutral version (pH 9.0) of the comet assay, was performed on cancer cell lines according to the procedures described by Singh et al. [60] with modifications [61,62]. 12-well plates were seeded at a density of 1.2–1.5 × 10^5^ cells per well. Subsequently, the cells were cultured for 24 h in controlled conditions (5% CO_2_; 37 °C), and then were exposed to investigated compounds in different concentrations (0.5 IC_50_, IC_50_, and 2 × IC_50_ values obtained in MTT assay). In addition, negative and positive controls (cells treated only with DMSO at <0.5% and with bleomycin at 20 µM, respectively) were included.

Following a 24 h incubation (5% CO_2_; 37 °C), the cells were trypsinized and transferred to Eppendorf tubes and centrifuged (10 min, 1400 rpm, 4 °C). The obtained pellet was resuspended in 100 µL of fresh PBS, mixed with 0.75% LMP agarose dissolved in PBS, and embedded onto microscope slides, previously precoated with 0.5% NMP agarose. The Trypan Blue dye exclusion test was used to determine the number of viable cells present in a cell suspension. Cell viability did not fall below the required 80%. The slides after agarose solidification were placed in a fresh, cold lysis buffer (pH 10, 1% Triton X-100, 2.5 M NaCl, 100 mM EDTA, 10 mM Tris) for 1 h (4 °C, in dark), which subsequently was replaced by electrophoretic solution (1 mM EDTA, 300 mM NaOH, pH > 13) for another 40 min. Afterward, electrophoresis was performed (25 min, 0.73 V/cm, 300 mA, 4 °C, in dark). Subsequently, the slides were neutralized with 0.4 M Tris, pH 7.5 (20 min, 4 °C, in dark) and washed 3× in distilled water. Eventually, slides were drained, stained with DAPI (1 μg/mL) for at least 1 h (4 °C), and analyzed with fluorescence microscope (Olympus, Tokyo, Japan).

The neutral version of the comet assay was conducted according to the same procedure as the alkaline counterpart with a few exceptions, including different electrophoretic buffer (100 mM Tris HCL, 300 mM sodium acetate, pH adjusted to 9.0 by glacial acetic acid), as well as different electrophoresis conditions (60 min; 4 °C; in dark; electric field strength of 0.41 V/cm (50 mA)).

A total number of 50 randomly selected images were analyzed in the commercially available software CaspLab. Two parallel experiments with aliquots of the same sample of cells were performed for a total of 100 cells. From the available parameters, the percentage of DNA in comet tail was selected as the most appropriate as being negatively associated with the level of DNA cross-links, as well as positively correlated with alkali-labile sites and the amount of DNA single-strand breaks (SSBs) and double-strand breaks (DSBs) (alkaline version of the comet assay). The same parameter was used as the measure of DSBs present in the neutral version of the comet assay [63,64,65].

#### 4.5.2. Immunocytochemical Detection of γ-H2AX

Immunocytochemical detection of the phosphorylated form of the histone protein component H2AX (γ-H2AX) is a well-known approach to estimate DNA DSB levels in both in vitro [47,53,66] and in vivo [67,68] studies. HCT 116 cells were grown on coverslips in 12-well plates at a density of 1.2 × 10^5^ per well for 24 h (5% CO_2_; 37 °C). Subsequently, the cells were exposed to investigated compounds in two different concentrations (IC_50_ and 2 × IC_50_ values with respect to MTT results), by replacing the medium with an appropriate compound concentration. Negative control (cells treated only with DMSO at <0.5%) was also included.

The cells were fixed with 4% paraformaldehyde (10 min), washed twice with PBS, and permeabilized with 0.5% (*v*/*v*) Triton X-100/PBS solution (10 min). Afterward, the cells were incubated with a blocking solution containing 1.5% goat serum, and 0.1% Triton in PBS for 10 min and incubated with the primary antibody, anti-gamma-H2AX (phospho-Ser139) (Abcam, Cambridge, UK; 1:100) for 1 h. The cells were washed with PBS and were incubated for another 1 h with the secondary Goat anti-Mouse IgG Cross-adsorbed antibody, Alexa Fluor 488 (Invitrogen; Carlsbad, CA, USA, 1:500). Following incubation with a secondary antibody, the cells were additionally stained with DAPI (2 μg/mL) in PBS (10 min). Finally, the cells were covered with slides in Fluoromount-G (Invitrogen, Carlsbad, CA, USA) mounting medium.

The slides were examined using a fluorescence microscope (Olympus) at 40× magnification. The commercially available software ImageJ was used to analyze the mean number of γ-H2AX foci per nucleus in each sample. A total number of 150 randomly selected cells from digital images per sample were measured. The data were exported and analyzed in GraphPad Prism 8.0 software system (GraphPad Prism Software Inc., San Diego, CA, USA).

### 4.6. Statistical Analysis

All data are presented as mean ± 95% confidence intervals (MTT assay) or median with interquartile range and minimal and maximal values (comet assay and immunocytochemical detection of γ-H2AX). The results were obtained from three independent experiments. Statistical significance between the results was evaluated in GraphPad Prism 8.0 software system (GraphPad Prism Software Inc., San Diego, CA, USA), by using the Kruskal–Wallis test with Dunn’s test (multiple comparisons in distributions departing from normality). A *p*-value of < 0.05 was considered to be significant.

## 5. Conclusions

The current study shows for the first time the diverse, cell-line-dependent cytotoxicity and genotoxicity of **MM129**, **MM130** and **MM131** on HeLa, PC-3, HCT 116, and BxPC-3 cell lines. The MTT assay and the comet assay used in the alkaline and neutral versions, as well as immunocytochemical detection of γ-H2AX, proved high anticancer properties of **MM129**, **MM130** and **MM131** in vitro. Among all the examined MM compounds, **MM131** exhibited the strongest cytotoxic and genotoxic properties. Further investigations of biological activities of tested compounds, including their ability to induce oxidative stress and apoptosis, as well as their implications in the cell cycle progression, are currently being investigated. Nevertheless, our present results constitute a foundation for further in vivo studies, which may lead to the selection of the most efficient compounds among the group of de novo synthesized pyrazolo[4,3-*e*]tetrazolo[1,5-*b*][1,2,4]triazine sulfonamides.

## Figures and Tables

**Table 1:** Comparison of the results of the alkaline and the neutral comet assay. DNA damage was shown as a median of the percentage of DNA in comet tail with an interquartile range (parentheses). Significant differences (*p* < 0.0001) compared with control (*).

Cancer Cell Line	Compound	Alkaline Version	Neutral Version
HCT 116	Control	1.3 (0.5–2.1)	2.9 (1.9–4)
	**MM129** IC_50_	10.7 * (4.7–28.1)	19.4 * (13.3–29.2)
	**MM129** 2 × IC_50_	24.9 * (12.4–46.3)	26.9 * (19.9–34.3)
	**MM130** IC_50_	22.7 * (10.3–44)	21.2 * (14.9–31.7)
	**MM130** 2 × IC_50_	25.6 * (14.1–41.3)	30.6 * (24.8–36.1)
	**MM131** IC_50_	31.5 * (19.8–51.3)	15.8 * (11.7–23.1)
	**MM131** 2 × IC_50_	38.8 * (25.3–57)	25.6 * (19.7–33.1)
PC-3	Control	3.4 (2.2–4.8)	1.3 * (0.4–2.3)
	**MM129** IC_50_	13.8 * (4–30.6)	2.4 * (1.1–3.8)
	**MM129** 2 × IC_50_	21.8 * (10.4–38.2)	7.3 * (5.1–10.3)
	**MM130** IC_50_	12.4 * (4.2–33.4)	1.5 * (0.4–3.1)
	**MM130** 2 × IC_50_	19.8 * (9.6–34)	2.2 * (1.2–3.7)
	**MM131** IC_50_	29.4 * (17.9–41)	2.3 * (1.3–3.6)
	**MM131** 2 × IC_50_	40.3 * (28–53.2)	4.3 * (2.8–6.3)
BxPC-3	Control	1.6 * (0.5–2.6)	2.5 * (0.4–4.4)
	**MM129** IC_50_	6.7 * (2.3–20.7)	8.7 * (6.1–13.9)
	**MM129** 2 × IC_50_	20.7 * (10.5–27.4)	10 * (6.9–13.9)
	**MM130** IC_50_	9.1 * (2.2–25)	7 * (4.7–12.2)
	**MM130** 2 × IC_50_	15.8 * (4.5–29.9)	8.5 * (6.1–12.9)
	**MM131** IC_50_	7.5 * (1.7–23.9)	9.6 * (6.8–13.4)
	**MM131** 2 × IC_50_	17.4 * (2.5–27.7)	9.7 * (7.4–12.9)

## Data Availability

Not applicable.

## Supplementary Materials

The following supporting information can be downloaded at: https://www.mdpi.com/article/10.3390/molecules27123761/s1, Figure S1: 1H NMR in CDCl3 for derivative **1**; Figure S2: 1H NMR in CDCl3 for derivative **2a**; Figure S3: 1H NMR in acetone for derivative **2a**; Figure S4: 13C NMR in acetone for derivative **2a**; Figure S5: HRMS for derivative **2a**; Figure S6: 1H-NMR in DMSO for derivative **2b**; Figure S7: 1H-NMR for derivative **2b** in DMSO with one drop of D2O; Figure S8: 13C-NMR for derivative **2b**; Figure S9: HRMS for **2b**; Figure S10: 1H-NMR for derivative **2c**; Figure S11: 13C-NMR for derivative **2c**; Figure S12: HRMS for **2c**; Figure S13: 1H NMR in MeOH-d4 for derivative **MM129**; Figure S14: 13C NMR in MeOH-d4 for derivative **MM129**; Figure S15: 1H NMR in CDCl3 for derivative **MM129**; Figure S16: 1H-NMR in MeOH-d4 for derivative **MM130**; Figure S17: 13C NMR in MeOH-d4 for derivative **MM130**; Figure S18: 1H-NMR in MeOH-d4 for derivative **MM131**; Figure S19: 13C-NMR in MeOH-d4 for derivative **MM131**.

## Sample Availability

Samples of the compounds **MM129**, **MM130** and **MM131** available from the authors.
