# Supplementary material for: Pyrazolo[4,3-e]tetrazolo[1,5-b][1,2,4]triazine Sulfonamides as Novel Potential Anticancer Agents: Cytotoxic and Genotoxic Activities In Vitro
Source: Molecules. 2022 Jun 11;27(12):3761. doi: 10.3390/molecules27123761 (PMC9229263; doi:10.3390/molecules27123761)

molecules

Supplementary Materials

Pyrazolo[4,3-*e*]tetrazolo[1,5-*b*][1,2,4]triazine Sulfonamides as Novel Potential Anticancer Agents: Cytotoxic and Genotoxic Activities In Vitro

Karol Bukowski [1,*], Beata Marciniak [1], Mateusz Kciuk [1,2], Mariusz Mojzych [3] and Renata Kontek [1]

[1] Department of Molecular Biotechnology and Genetics, Faculty of Biology and Environmental Protection, University of Lodz, 12/16 Banacha St., 90-237 Lodz, Poland; beata.marciniak@biol.uni.lodz.pl (B.M.); mateusz.kciuk@edu.uni.lodz.pl (M.K.); renata.kontek@biol.uni.lodz.pl (R.K.)
[2] Doctoral School of Exact and Natural Sciences, University of Lodz, 12/16 Banacha St., 90-237 Lodz, Poland
[3] Department of Chemistry, Siedlce University of Natural Sciences and Humanities, 3 Maja 54, 08-110 Siedlce, Poland; mariusz.mojzych@uph.edu.pl
* Correspondence: karol.bukowski@edu.uni.lodz.pl

Figure S1: ^{1}H-NMR in $CDCl_3$ for derivative **1**

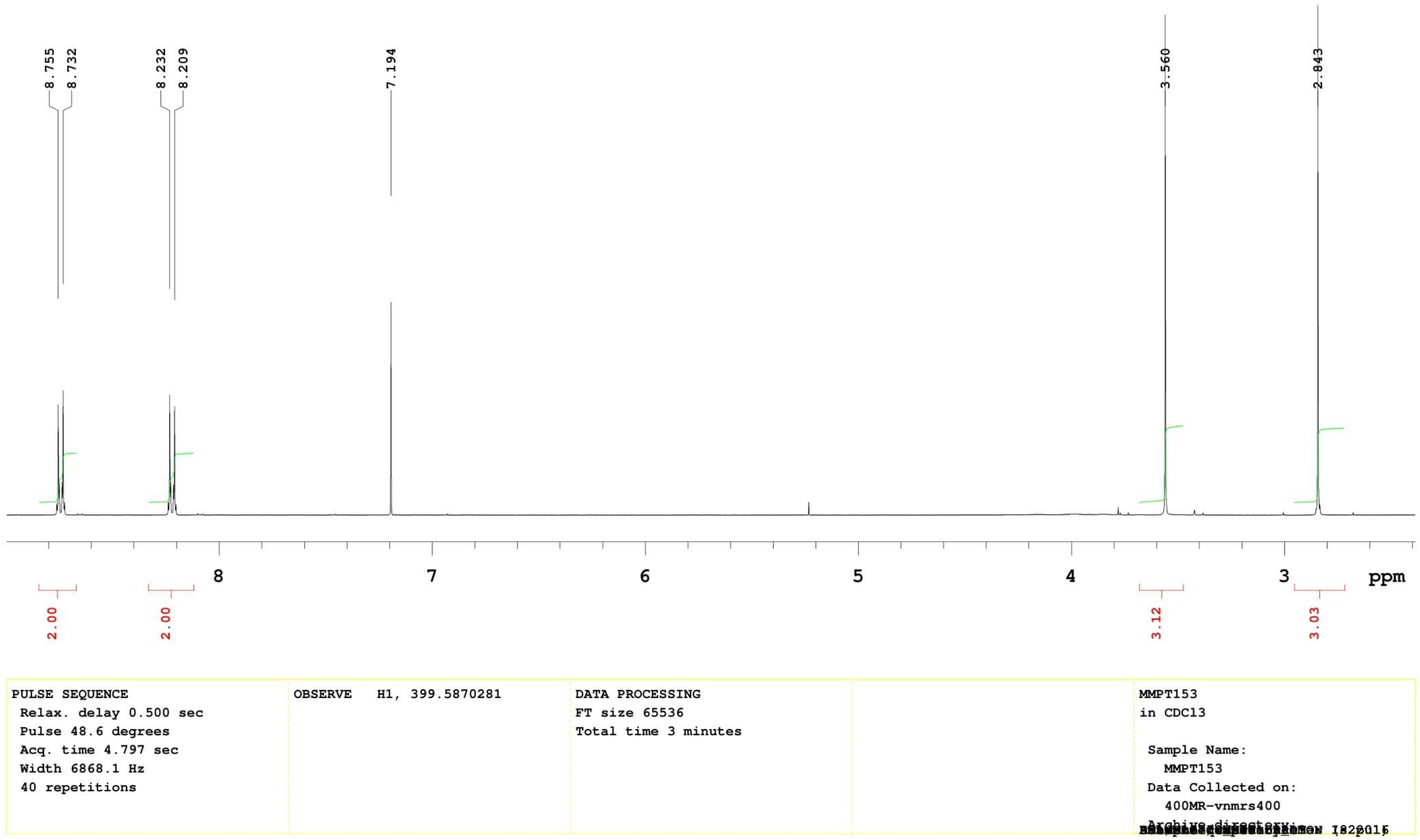

Figure S2: ^{1}H-NMR in $CDCl_3$ for derivative **2a**

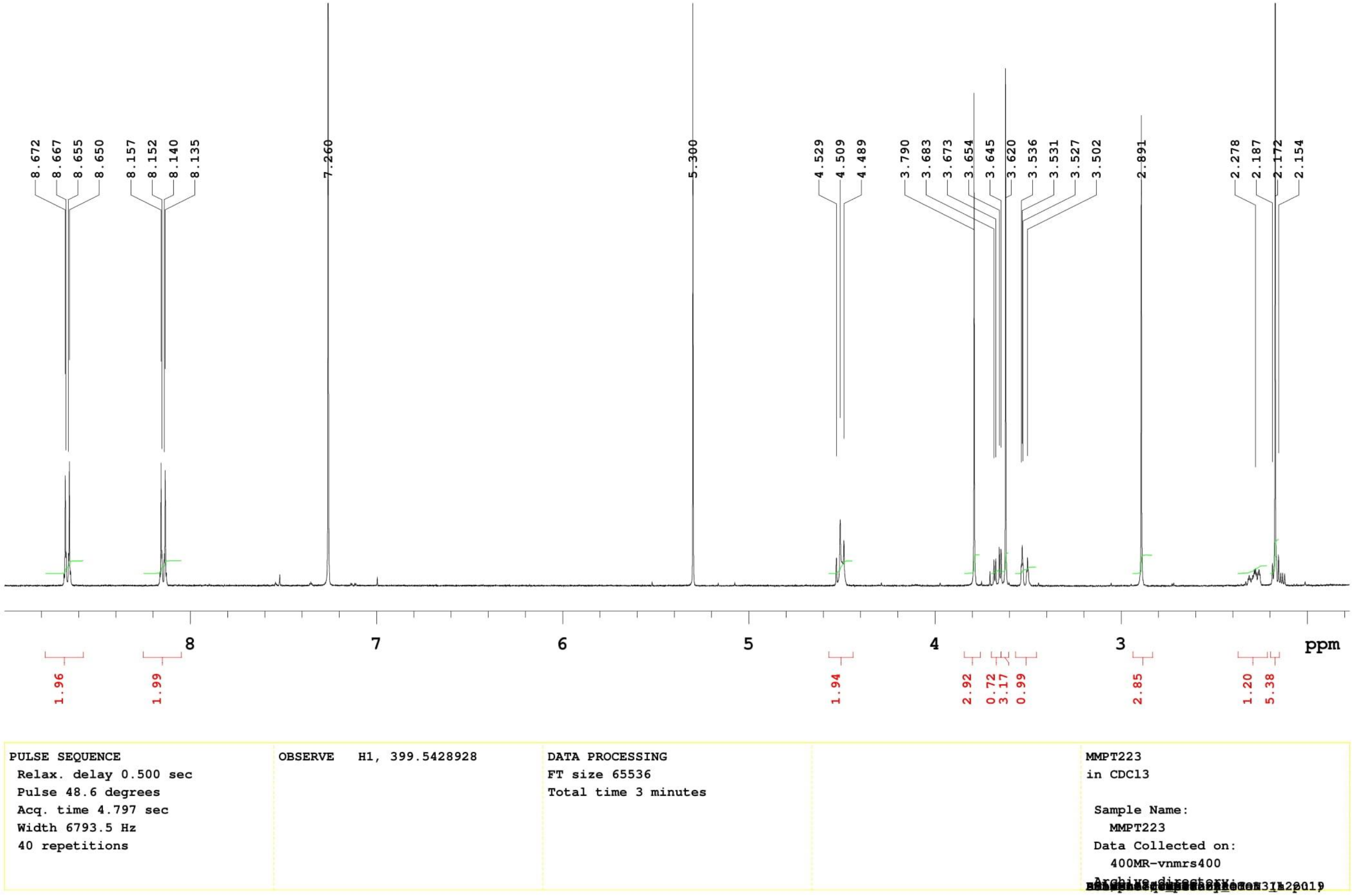

Figure S3: ^{1}H-NMR in acetone for derivative **2a**

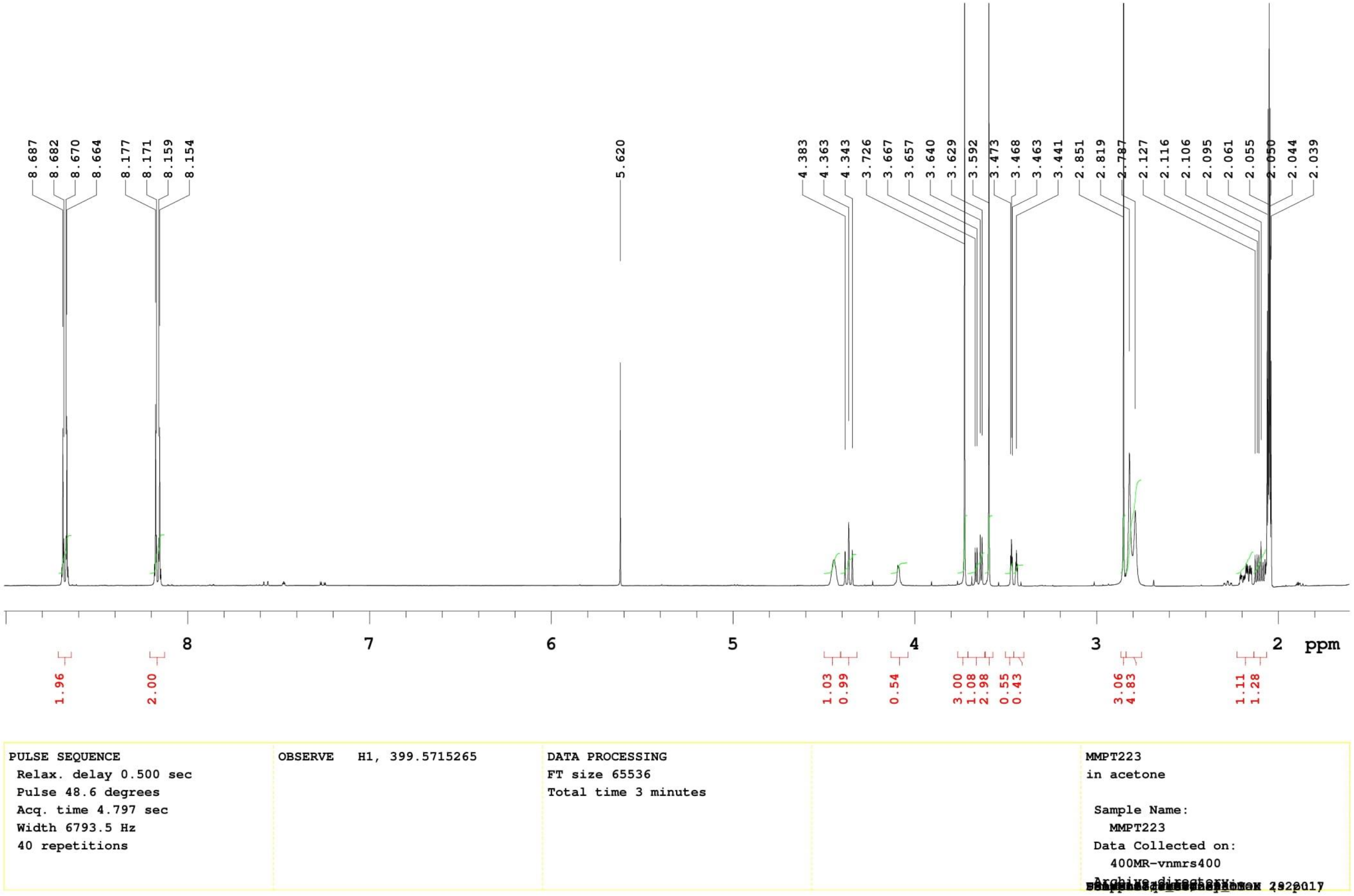

Figure S4: ^{13}C-NMR in acetone for derivative **2a**

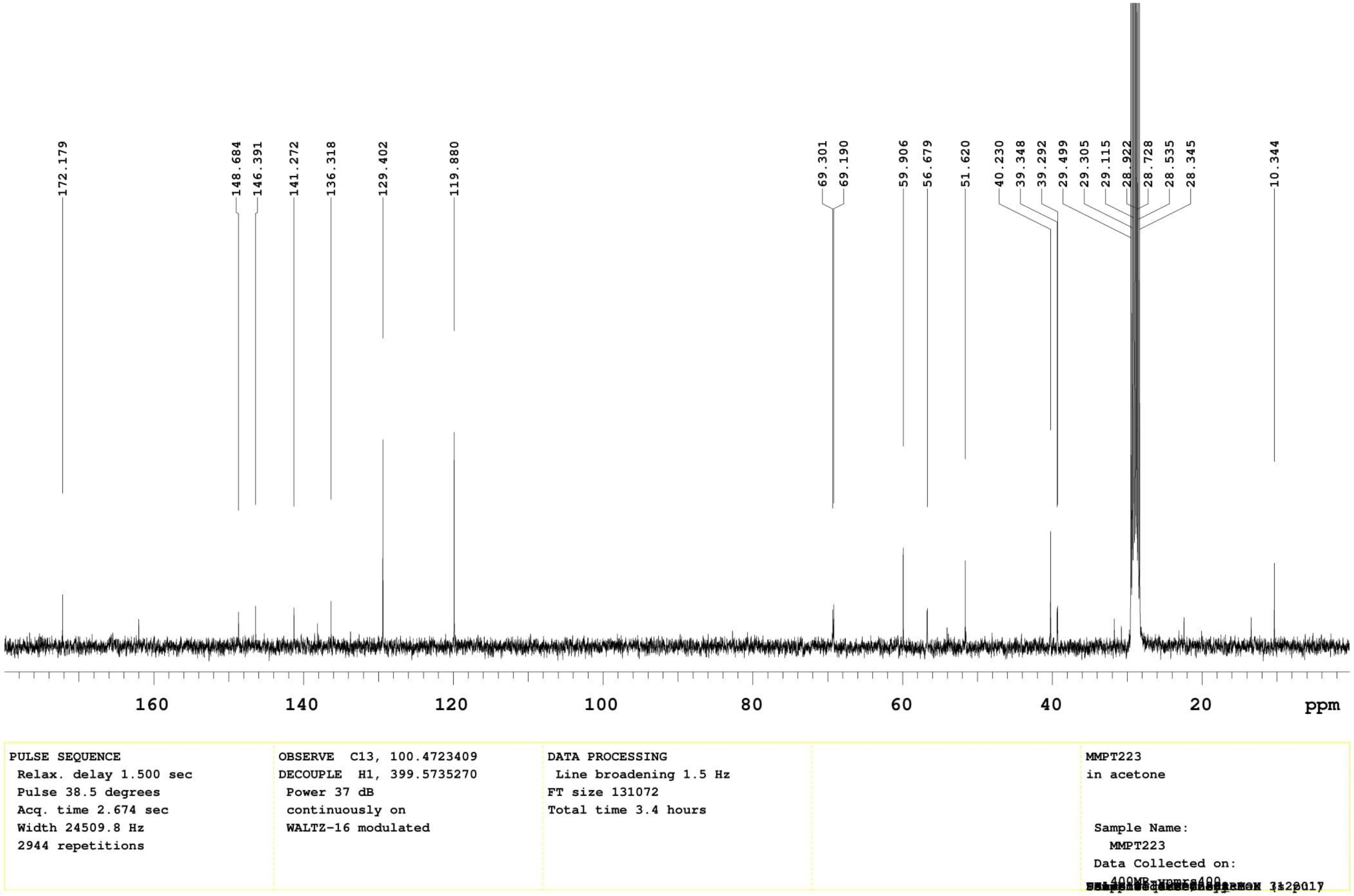

Figure S5: HRMS for derivative **2a**

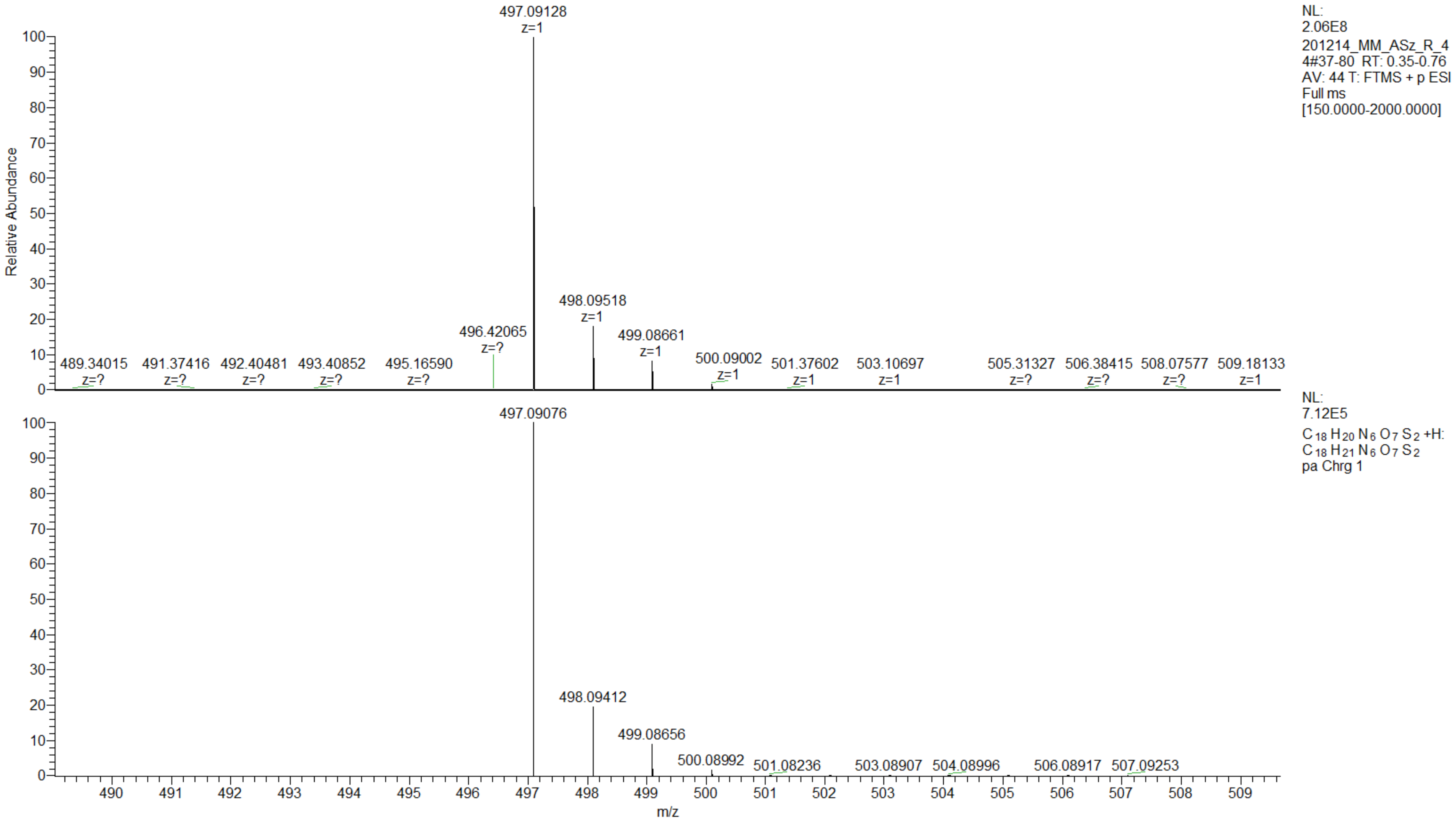

Figure S6: ^{1}H-NMR in DMSO for derivative **2b**

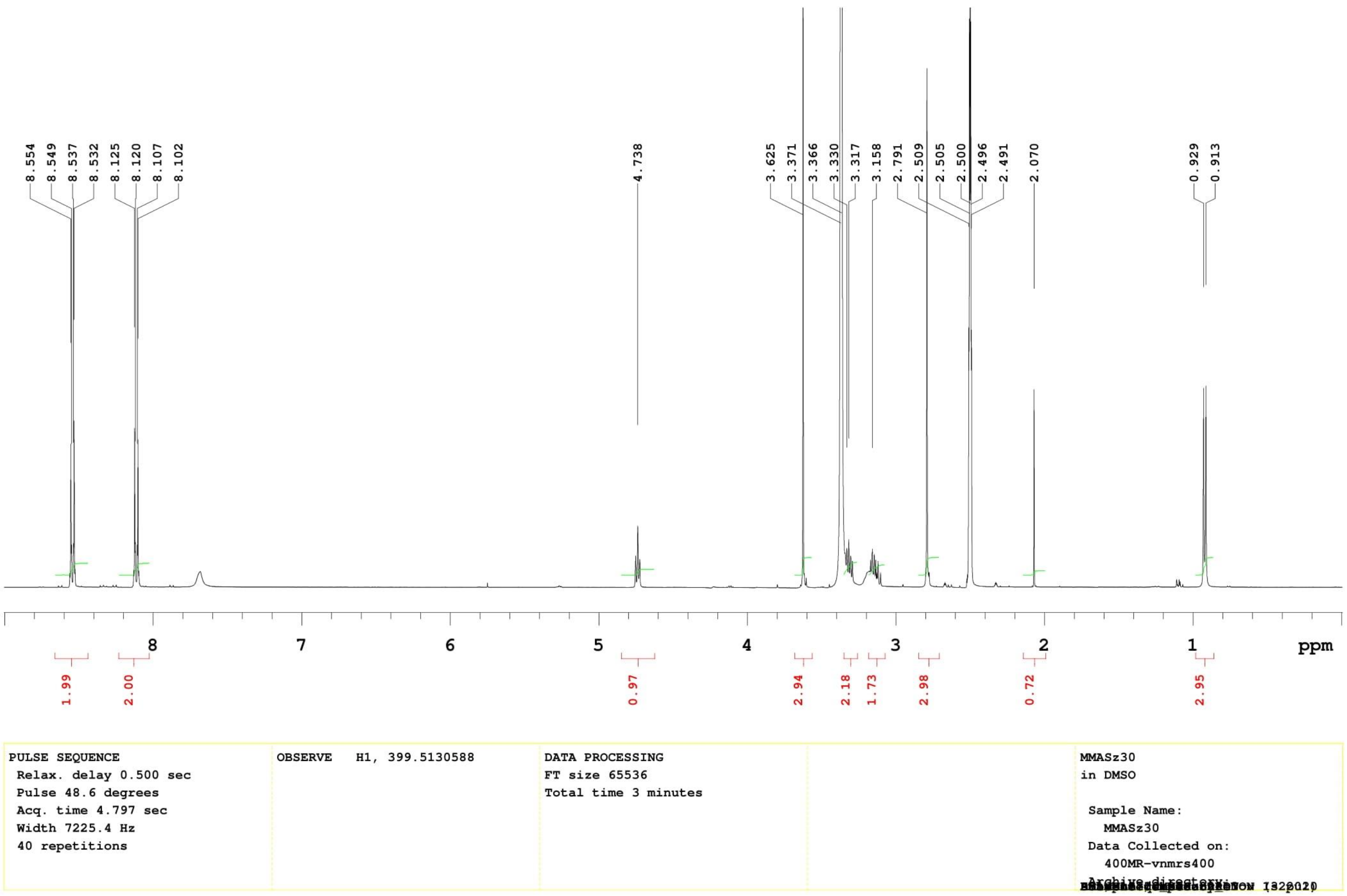

Figure S7: ^{1}H-NMR for derivative **2b** in DMSO with one drop of D_2O

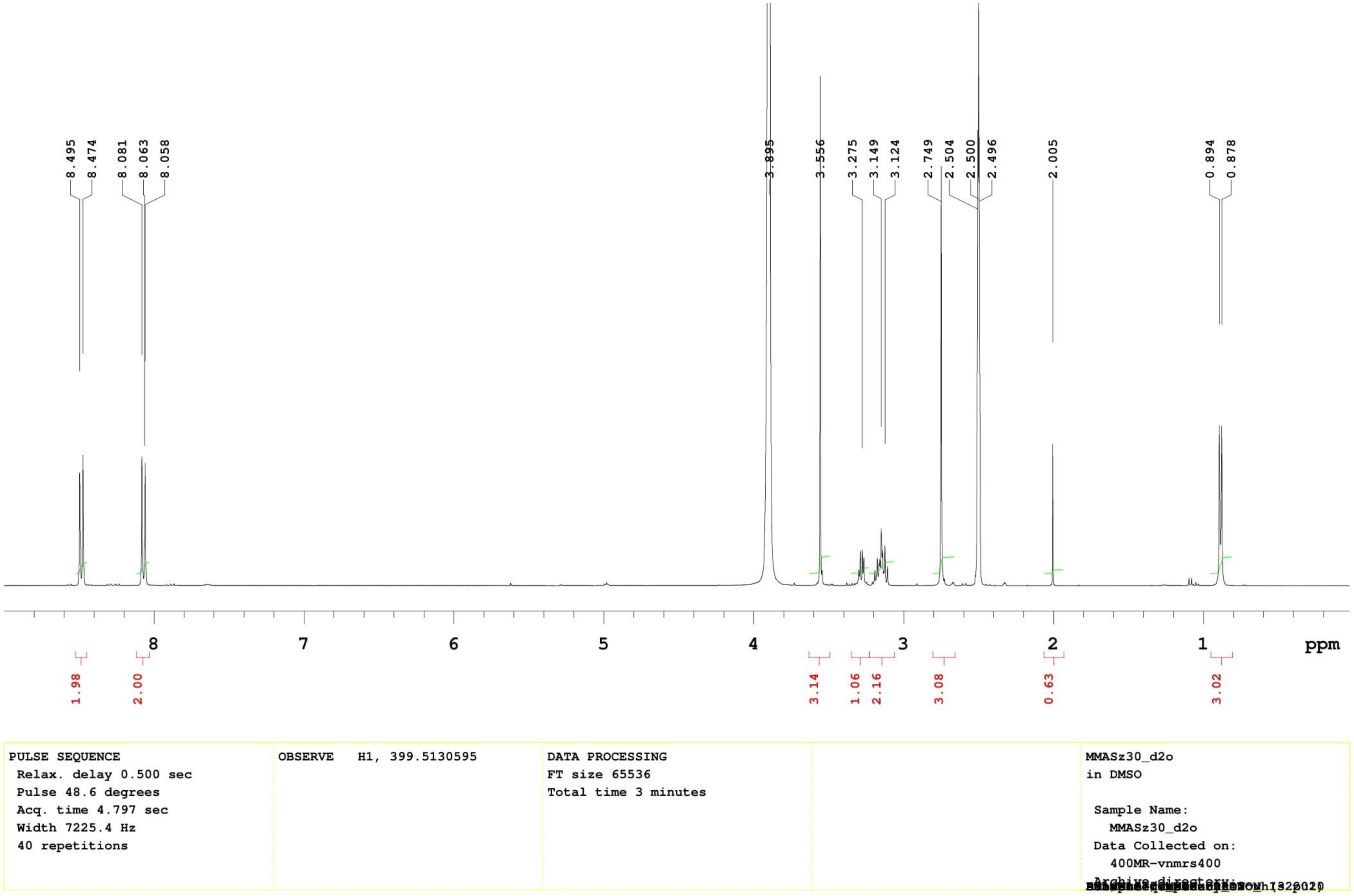

Figure S8: ^{13}C-NMR for derivative **2b**

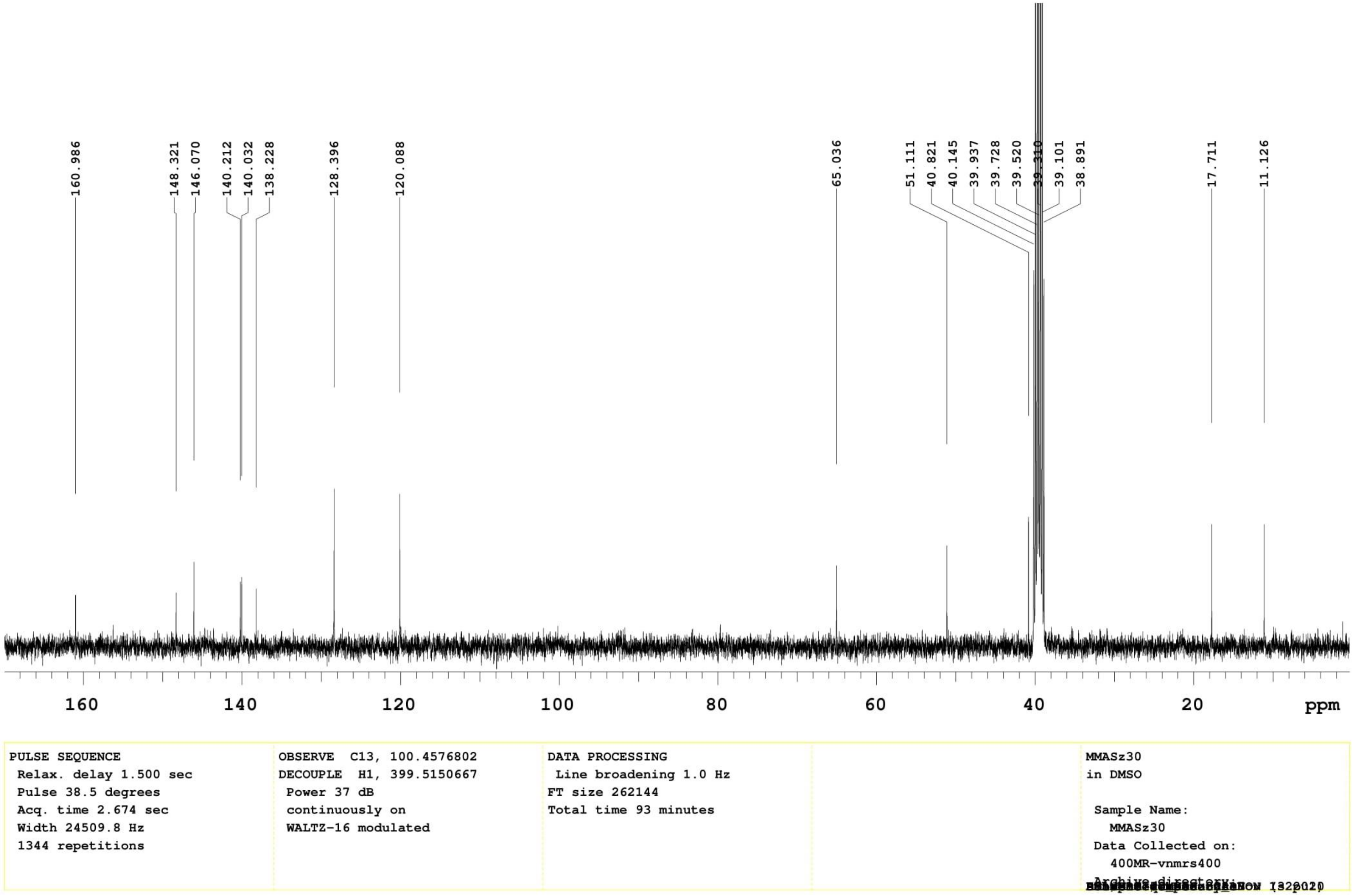

Figure S9: HRMS for **2b**

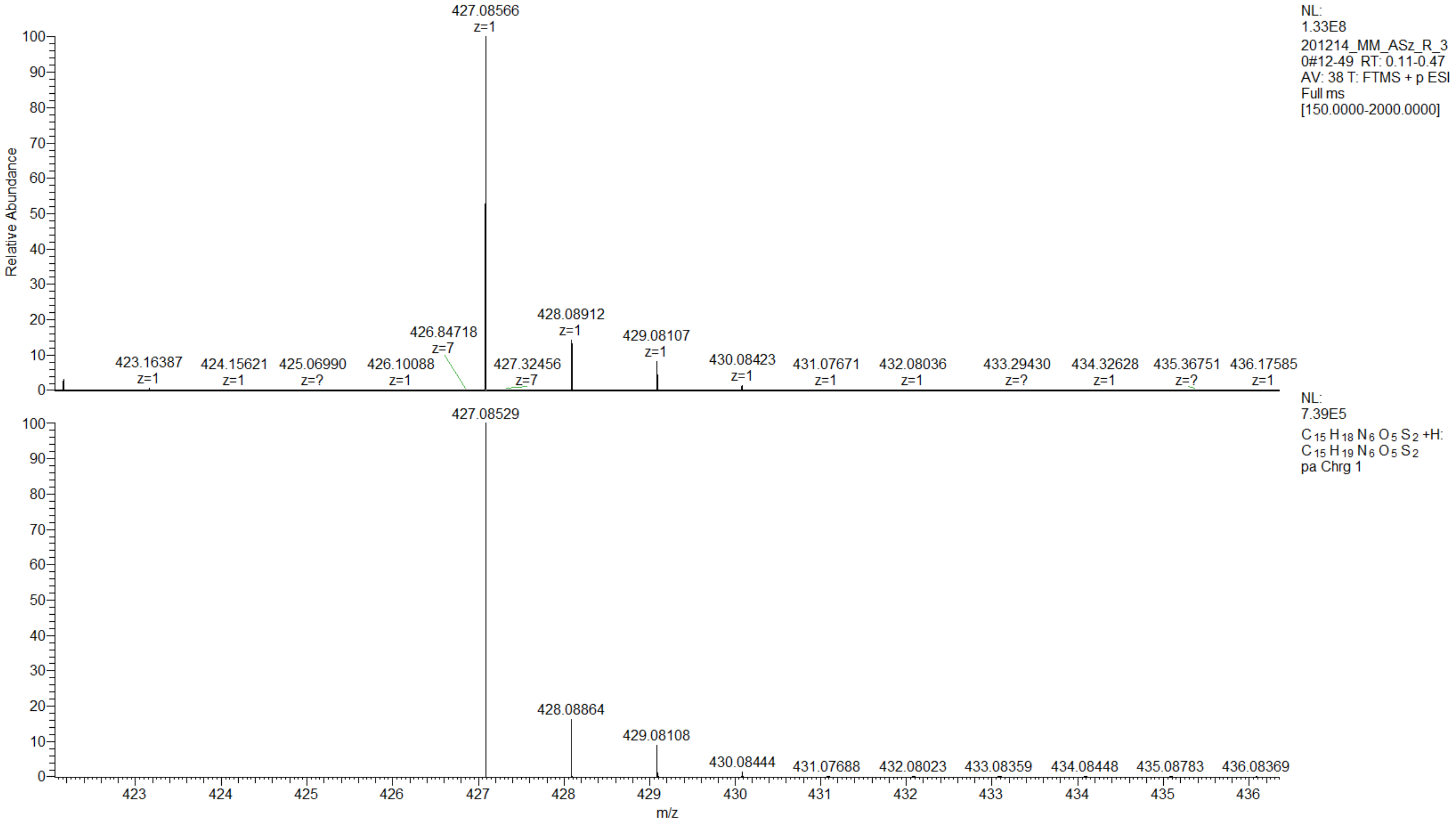

Figure S10: ^{1}H-NMR for derivative **2c**

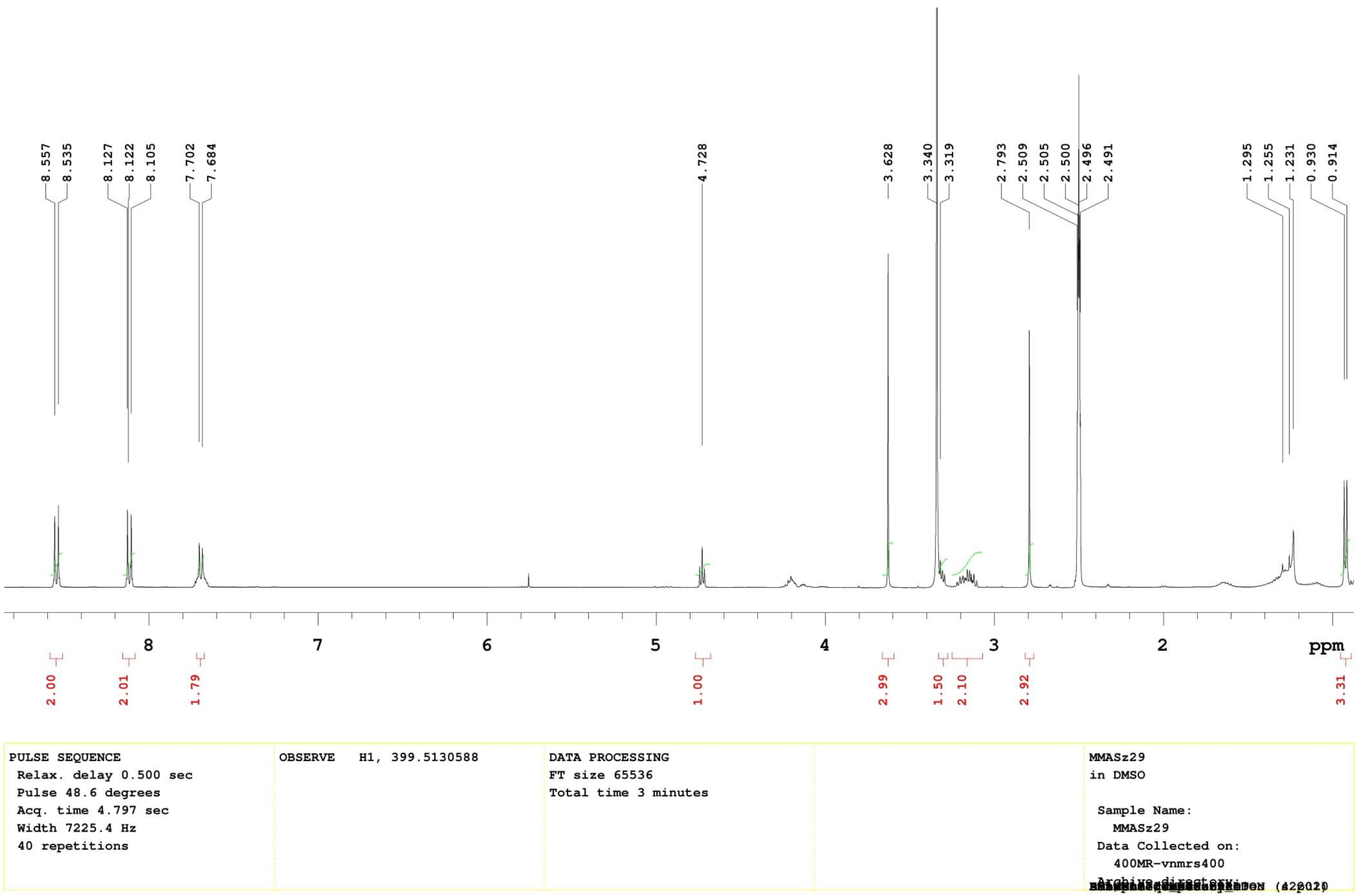

Figure S11: ^{13}C-NMR for derivative **2c**

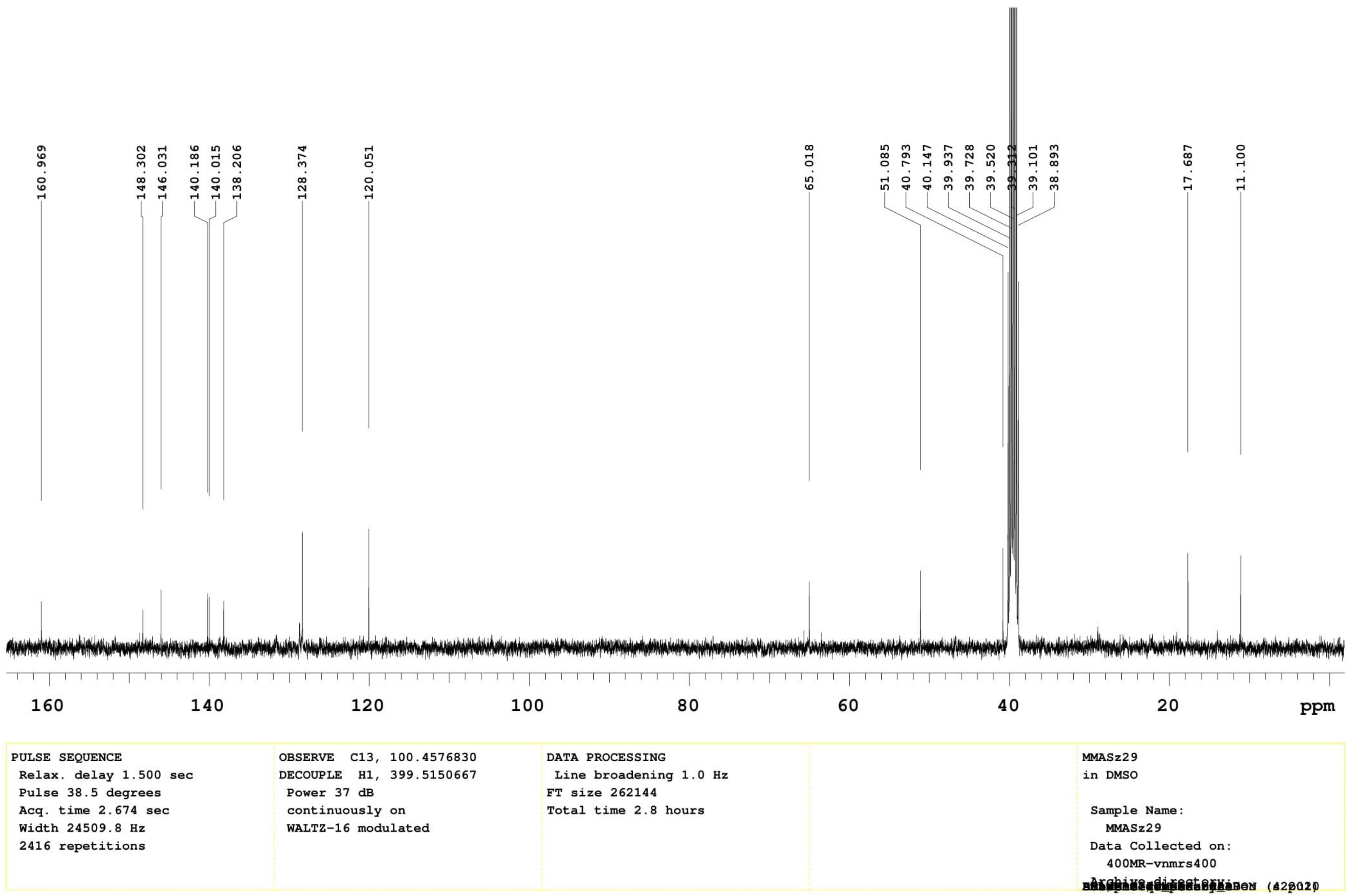

Figure S12: HRMS for **2c**

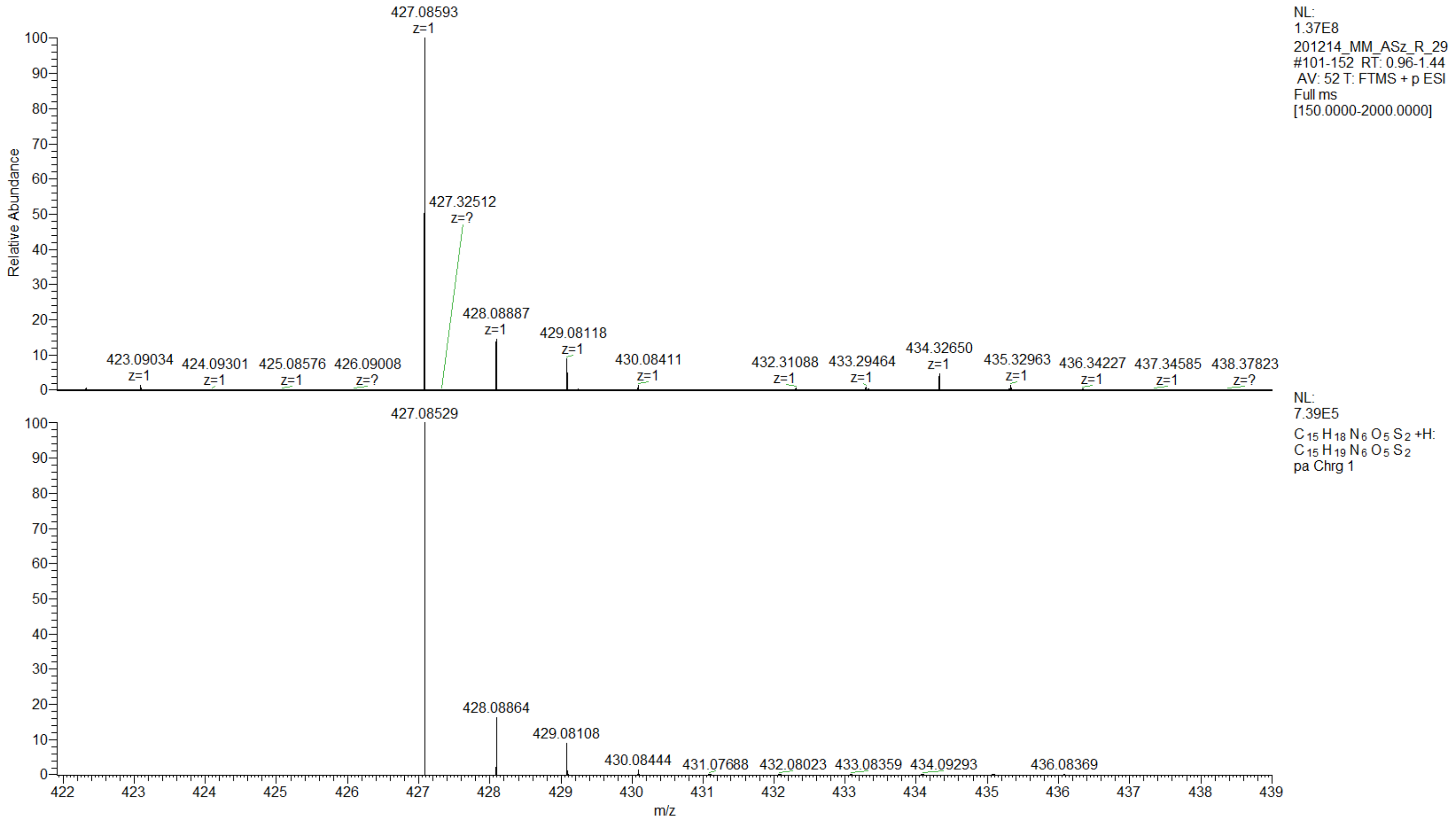

Figure S13: ^{1}H-NMR in MeOH-$_{d4}$ for derivative **MM129**

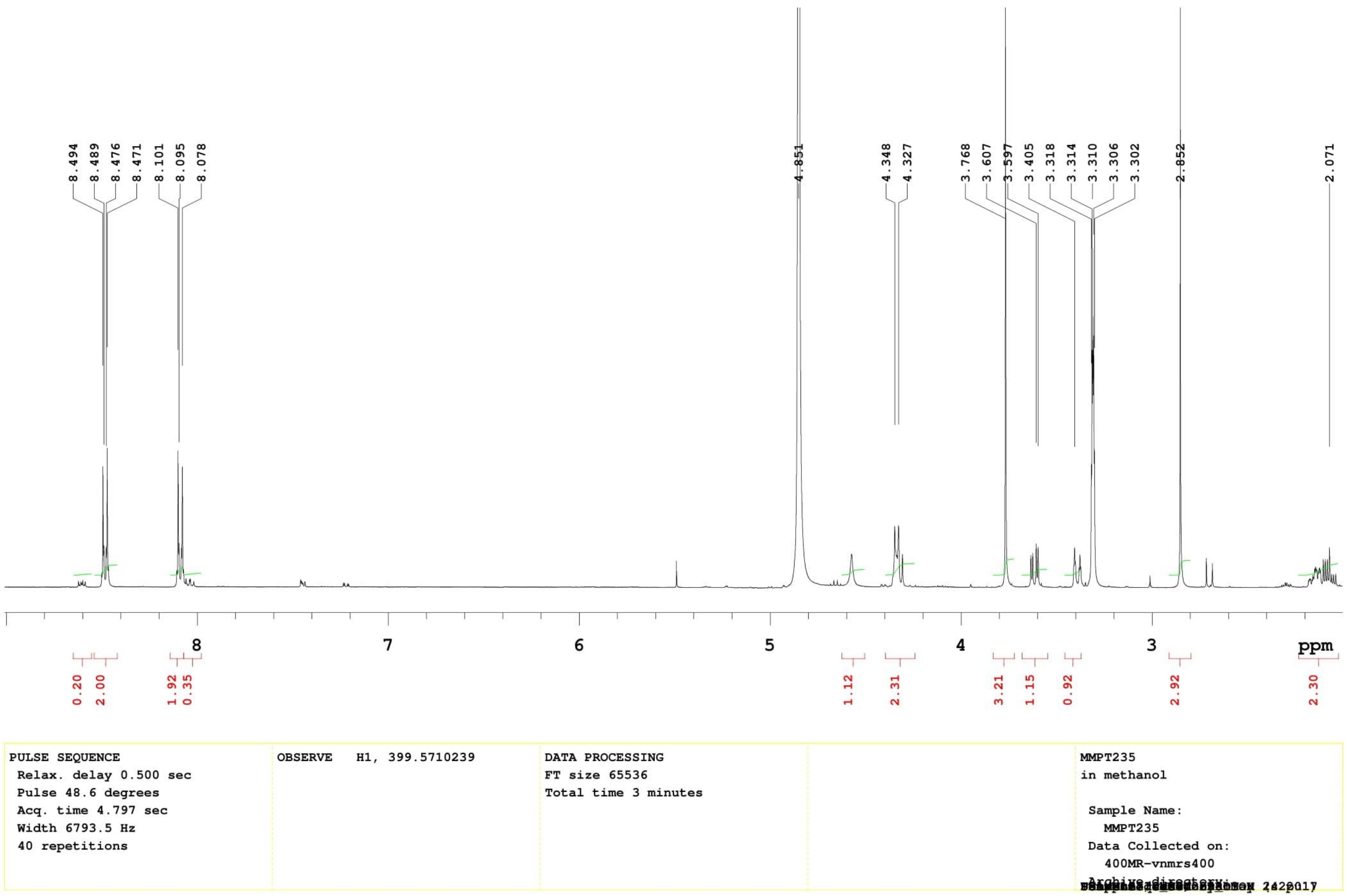

Figure S14: ^{13}C-NMR in MeOH-d4 for derivative **MM129**

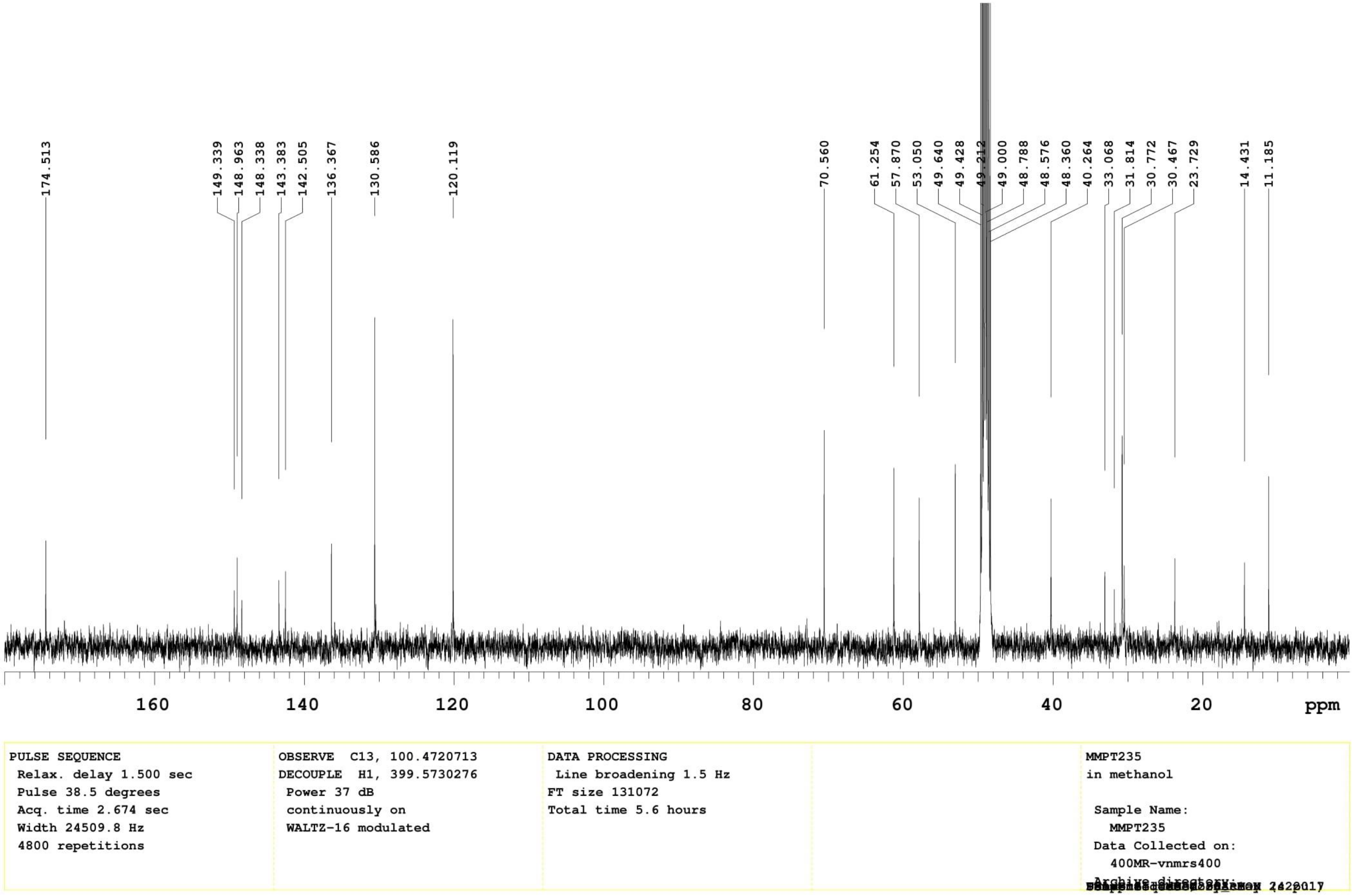

Figure S15: ^{1}H-NMR in $CDCl_3$ for derivative **MM129**

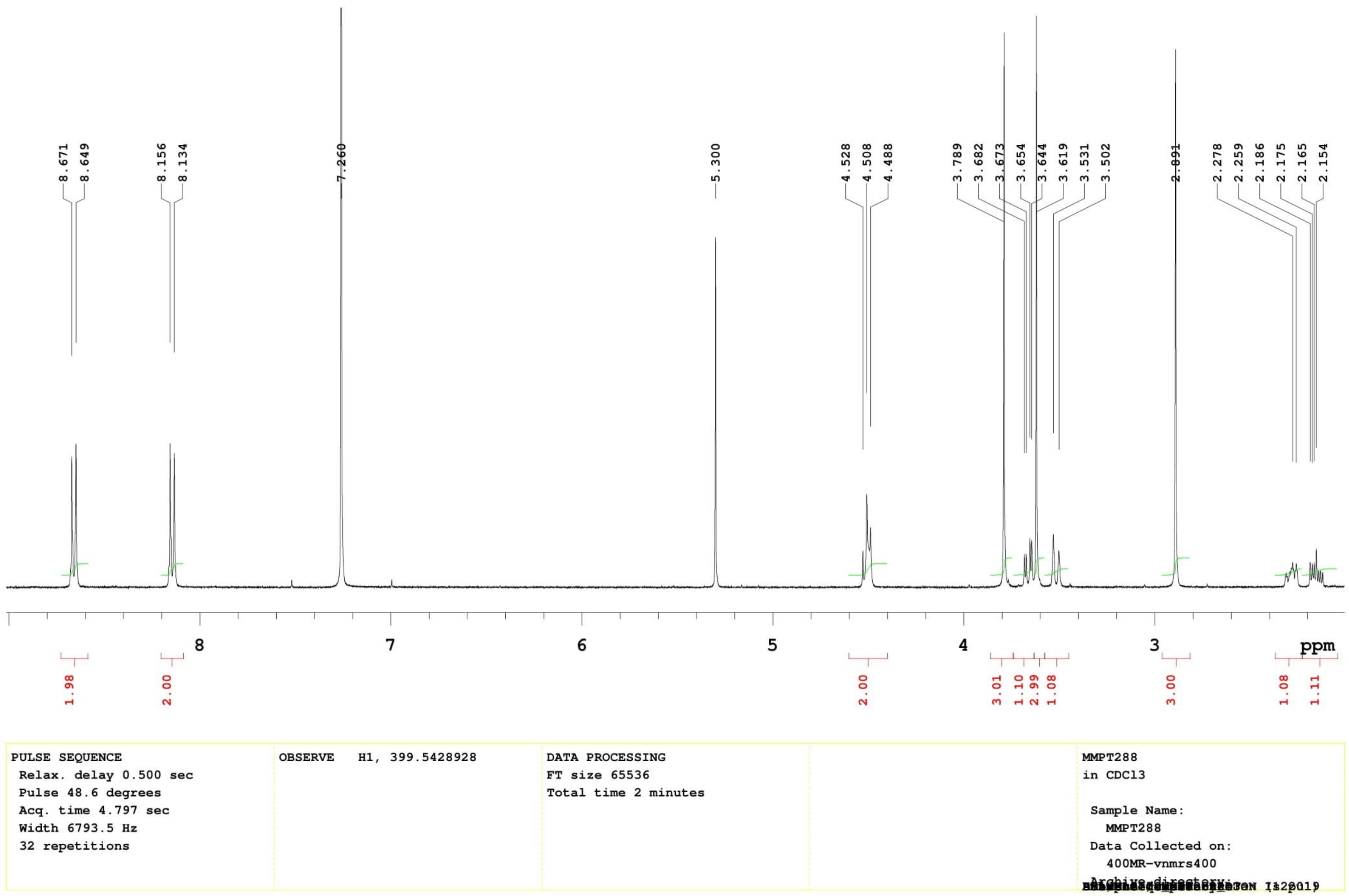

Figure S16: ^{1}H-NMR in MeOH-d4 for derivative **MM130**

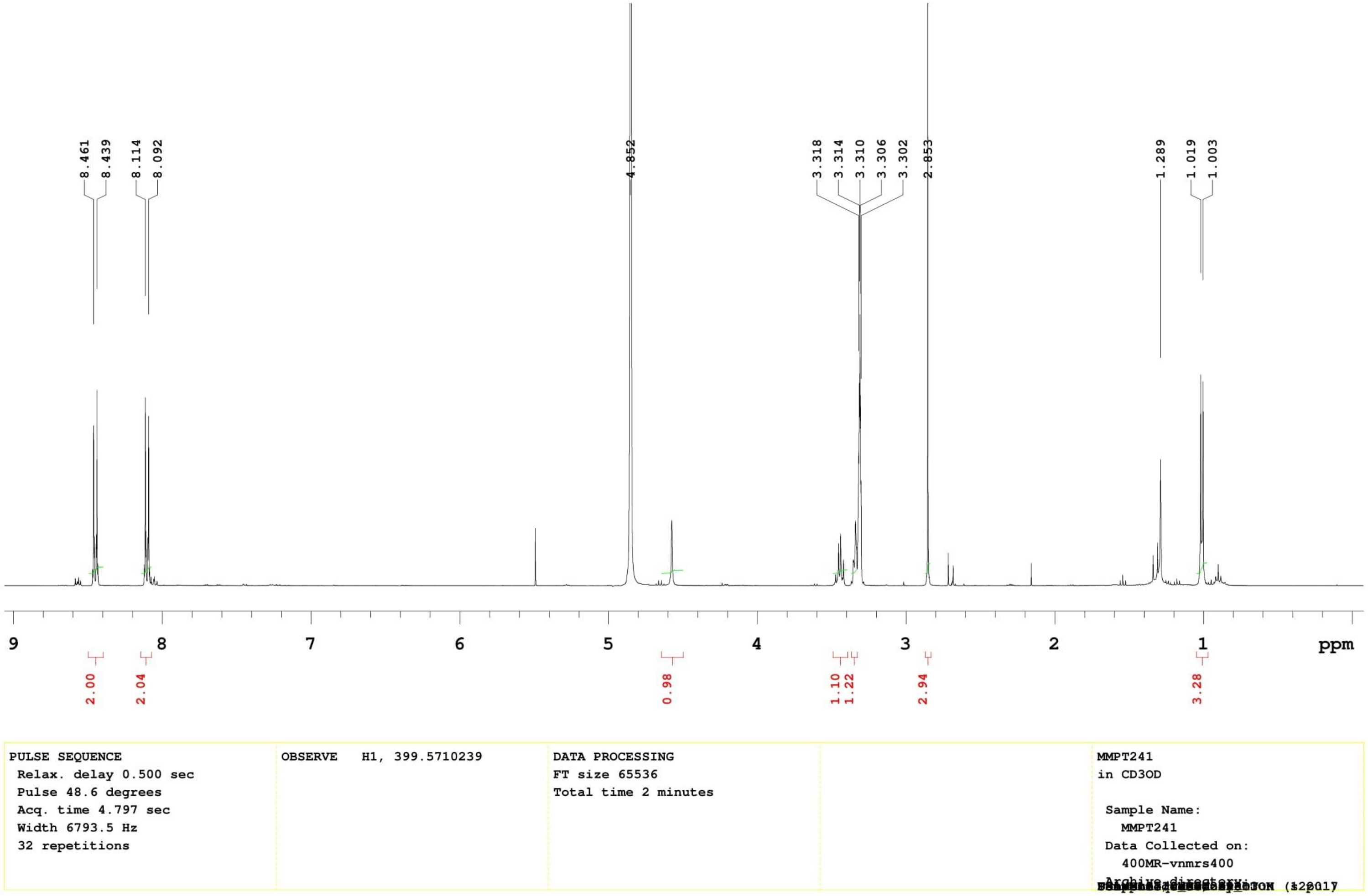

Figure S17: ^{13}C-NMR in MeOH-$_{d4}$ for derivative **MM130**

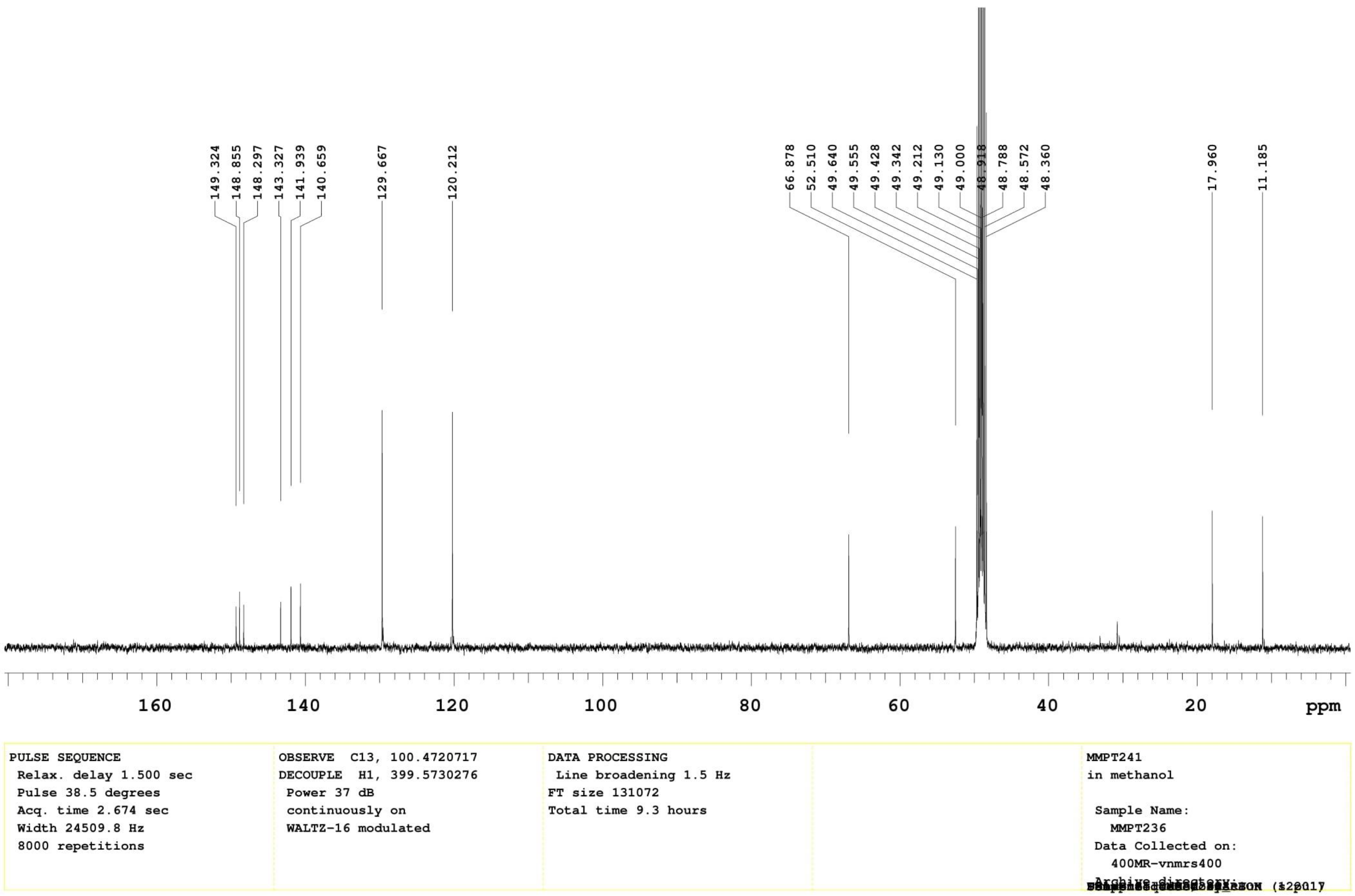

Figure S18: ^{1}H-NMR in MeOH-$_{d4}$ for derivative **MM131**

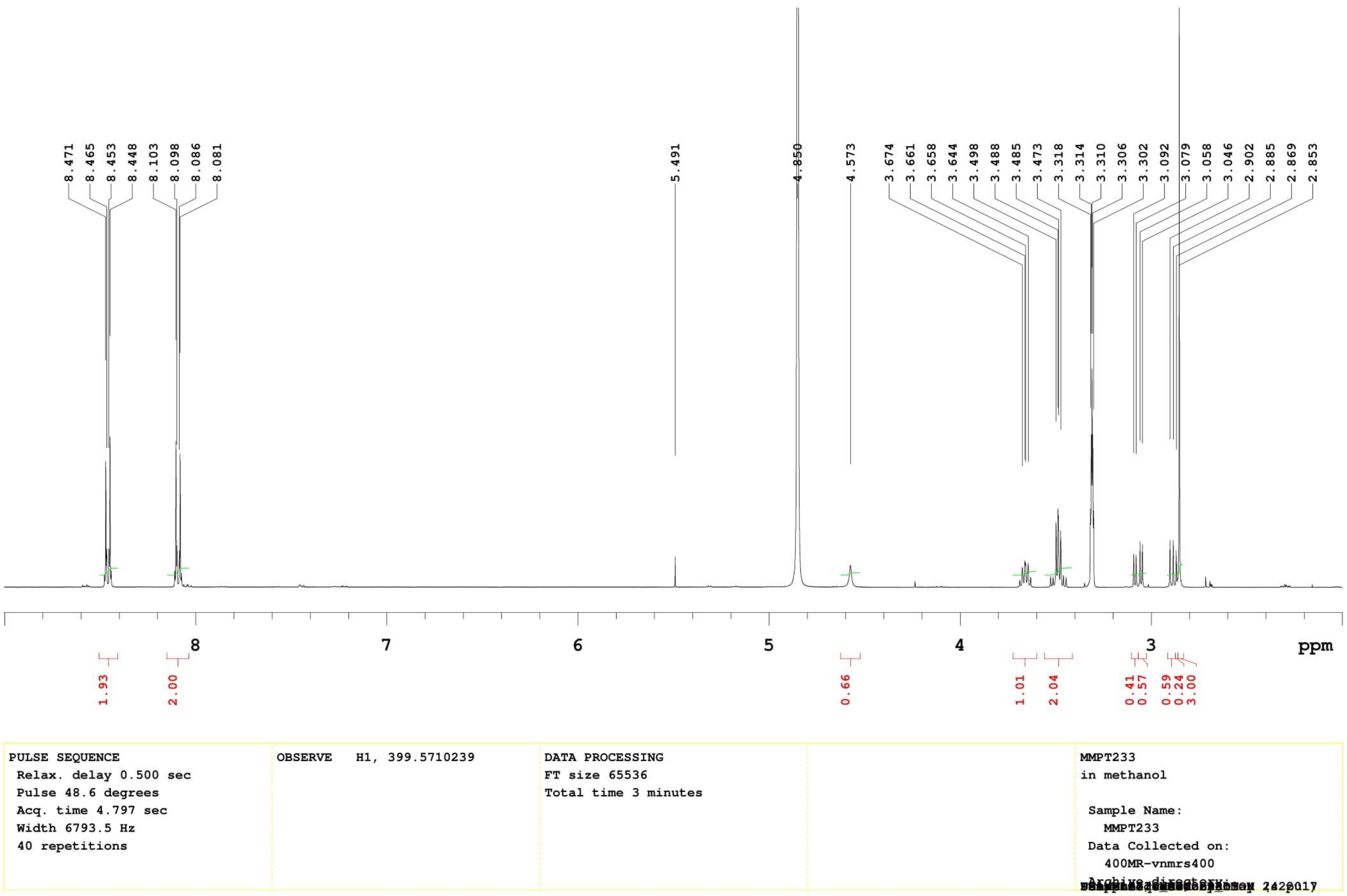

Figure S19: ^{13}C-NMR in MeOH-$_{d4}$ for derivative **MM131**

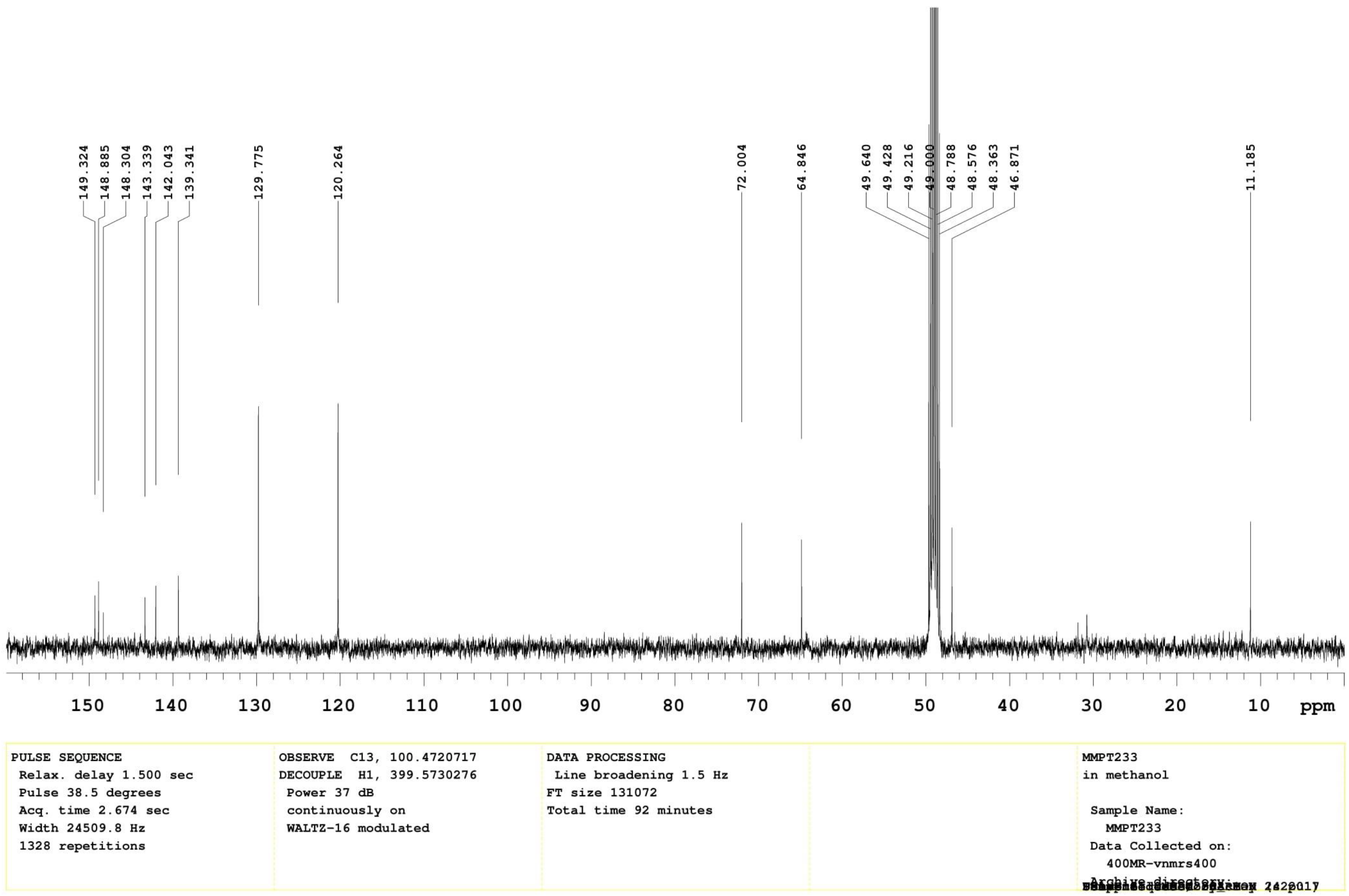

Supplement: Supplementary file 1 [file molecules-27-03761-s001.zip › molecules-1722448-supplementary.pdf]
